# Enhancer transcription profiling reveals an enhancer RNA-driven ferroptosis and new therapeutic opportunities in prostate cancer

**DOI:** 10.1038/s41392-025-02170-6

**Published:** 2025-03-14

**Authors:** Sheng Ma, Zixian Wang, Zezhong Xiong, Yue Ge, Meng-Yao Xu, Junbiao Zhang, Yuzheng Peng, Qin Zhang, Jiaxue Sun, Zirui Xi, Hao Peng, Wenjie Xu, Yanan Wang, Le Li, Chunyu Zhang, Zheng Chao, Baojun Wang, Xu Gao, Xu Zhang, Gong-Hong Wei, Zhihua Wang

**Affiliations:** 1 Department of Urology, Tongji Hospital, Tongji Medical College, Huazhong University of Science and Technology, 430030 Wuhan, Hubei China; 2 MOE Key Laboratory of Metabolism and Molecular Medicine and Department of Biochemistry and Molecular Biology of School of Basic Medical Sciences & Fudan University Shanghai Cancer Center, Shanghai Medical College of Fudan University, Shanghai, China; 3 Disease Networks Research Unit, Faculty of Biochemistry and Molecular Medicine & Biocenter Oulu, University of Oulu, Oulu, Finland; 4 Department of Urology, Chinese PLA General Hospital, Beijing, China; 5 Department of Urology, Changhai Hospital, Shanghai, China; 6 State Key Laboratory of Common Mechanism Research for Major Diseases, Suzhou Institute of Systems Medicine, Chinese Academy of Medical Sciences & Peking Union Medical College, 215123 Suzhou, Jiangsu China; 7 Taikang Tongji (Wuhan) Hospital, 420060 Wuhan, China

**Keywords:** Urological cancer, Tumour biomarkers, Drug development

## Abstract

Enhancer RNAs (eRNAs), a subclass of non-coding RNAs transcribed from enhancer regions, have emerged as critical regulators of gene expression; however, their functional roles in prostate cancer remain largely unexplored. In this study, we performed integrated chromatin accessibility and transcriptomic analyses using ATAC-seq and RNA-seq on twenty pairs of prostate cancer and matched benign tissues. By incorporating chromatin immunoprecipitation sequencing data, we identified a subset of differentially expressed eRNAs significantly associated with genes involved in prostate development and oncogenic signaling pathways. Among these, lactotransferrin-eRNA (*LTF*e) was markedly downregulated in prostate cancer tissues, with functional analyses revealing its tumor-suppressive role. Mechanistically, *LTF*e promotes the transcription of its target gene, lactotransferrin (*LTF*), by interacting with heterogeneous nuclear ribonucleoprotein F (HNRNPF) and facilitating enhancer-promoter chromatin interactions. Furthermore, we demonstrate that the *LTF*e-*LTF* axis facilitates ferroptosis by modulating iron transport. Notably, androgen receptor (AR) signaling disrupts *LTF*e-associated chromatin looping, leading to ferroptosis resistance. Therapeutically, co- administration of the AR inhibitor enzalutamide and the ferroptosis inducer RSL3 significantly suppressed tumor growth, offering a promising strategy for castration-resistant prostate cancer. Collectively, this study provides novel insights into the mechanistic role of eRNAs in prostate cancer, highlighting the *LTF*e-*LTF* axis as a critical epigenetic regulator and potential therapeutic target for improved treatment outcomes.

## Introduction

Prostate cancer represents a significant global health challenge, ranking as the second most commonly diagnosed malignancy and the fifth leading cause of cancer-related mortality among men worldwide.^1^ The clinical course of prostate cancer is highly heterogeneous, ranging from indolent, slow-growing tumors to aggressive, metastatic disease with poor prognosis, necessitating precise molecular characterization to optimize treatment strategies. Early-stage, localized prostate cancer can often be managed effectively through radical prostatectomy, radiotherapy, and androgen deprivation therapy (ADT). However, despite these interventions, a substantial subset of patients still experiences disease recurrence and progression to castration-resistant prostate cancer (CRPC), a lethal form of the disease that arises when tumor cells acquire the ability to proliferate despite systemic androgen depletion.^2^ CRPC is characterized by the persistent activation of androgen receptor (AR) signaling, despite pharmacological suppression with AR-targeted agents such as enzalutamide and abiraterone. Over the past decade, large-scale genomic and transcriptomic profiling of prostate tumors has provided valuable insights into the molecular mechanisms driving tumor progression and therapy resistance.^3–5^ While these studies have significantly advanced our understanding of genetic alterations in Prostate cancer, relatively little is known about the contribution of non-coding regulatory elements, particularly enhancers, in disease progression. Emerging evidence suggests that enhancer dysregulation plays a central role in driving oncogenic transcriptional programs,^6,7^ highlighting the need for deeper exploration of enhancer-associated molecular mechanisms in prostate cancer biology.

Recent research has increasingly underscored the critical role of enhancer reprogramming and enhancer RNAs (eRNAs) in cancer progression.^8^ Enhancers are distal regulatory elements that fine-tune gene expression by interacting with gene promoters through chromatin looping, often in a cell type-specific manner.^9,10^ A subset of these enhancers produces bidirectionally transcribed non-coding RNAs, known as eRNAs, which have emerged as key regulators of transcriptional dynamics.^11^ Unlike other non-coding RNAs such as long non-coding RNAs (lncRNAs) and microRNAs (miRNAs), eRNAs lack generally lack polyadenylation and are typically produced bidirectionally in response to transcription factor binding.^12^ Recent studies have highlighted the ability of eRNAs to modulate gene expression through multiple mechanisms, including the stabilization of enhancer-promoter looping, the establishment of chromatin accessibility and recruitment of transcriptional coactivators, and the facilitation of release for the negative elongation factor (NELF) complex, ultimately modulating transcriptional activity.^13,14^ In hormone-driven cancers, such as breast and prostate cancer, eRNAs have been implicated in AR- and estrogen receptor (ER)-mediated transcriptional programs.^15^ Specifically, in prostate cancer, AR-driven enhancer networks orchestrate oncogenic gene expression, yet the functional contributions of eRNAs within these regulatory landscapes remain poorly understood.^13,14^ Given the strong association between enhancer activity and aggressive tumor phenotypes, investigating eRNA-mediated transcriptional control may provide novel insights into prostate cancer progression and therapeutic resistance.

Advances in chromatin profiling techniques, such as assay for transposase-accessible chromatin sequencing (ATAC-seq), has significantly advanced our understanding of chromatin accessibility, especially in clinically available or frozen tissue samples.^16^ ATAC-seq enables the identification of open chromatin regions, offering a powerful tool for mapping active enhancers in cancer cells and clinical samples. Genome-wide chromatin accessibility studies across various cancers, leveraging transcriptomic datasets from The Cancer Genome Atlas (TCGA) and other large-scale prostate cancer cohorts has uncovered widespread chromatin remodeling events associated with disease progression.^17^ However, the precise regulatory mechanisms governing chromatin accessibility in prostate tumorigenesis, particularly the role of eRNAs in modulating transcription, remain largely unexplored. Addressing these gaps is essential for understanding how epigenetic reprogramming contributes to prostate cancer malignancy and resistance to therapy.

To address this gap, we conducted in this study an integrated epigenetic analysis combining ATAC-seq and RNA-seq on twenty pairs of prostate cancer and matched adjacent normal tissues. This approach enabled us to identify eRNAs with potential functional relevance in prostate cancer pathogenesis. Among the candidates, lactotransferrin-eRNA (*LTF*e) emerged as a key tumor suppressive eRNA, exhibiting significantly reduced expression in prostate cancer tissues compared to normal controls. Functional assays revealed that LTFe inhibits prostate cancer cell proliferation and enhances ferroptosis, a form of iron-dependent cell death, by regulating the expression of its downstream target gene, lactotransferrin (*LTF*). Mechanistically, *LTF*e enhances *LTF* transcription by binding to heterogeneous nuclear ribonucleoprotein F (HNRNPF), a known RNA-binding protein, and facilitating enhancer-promoter interactions thereby promoting *LTF* transcription. Notably, we discovered that AR signaling disrupts these chromatin loops, leading to reduced *LTF*e and *LTF* expression, thereby attenuating ferroptosis. These findings suggest that targeting AR signaling could restore *LTFe* function and enhance ferroptotic sensitivity in prostate cancer cells. Furthermore, we demonstrated that the combination of the anti-AR drug enzalutamide (ENZ) and the ferroptosis inducer RSL3 synergistically suppresses prostate tumor growth, offering a promising therapeutic strategy for CRPC. Given the limited efficacy of current therapies in overcoming CRPC progression, our study provides compelling evidence that targeting eRNA-mediated enhancer regulation may serve as a novel approach to enhance ferroptosis and mitigate treatment resistance. Collectively, our findings illuminate a previously unrecognized role of LTFe in prostate cancer biology, providing a foundation for future studies exploring enhancer-based therapeutic strategies in aggressive prostate malignancies.

## Results

### The transcriptional landscape and characterization of enhancer transcription in human prostate cancer

**Fig. 1:**
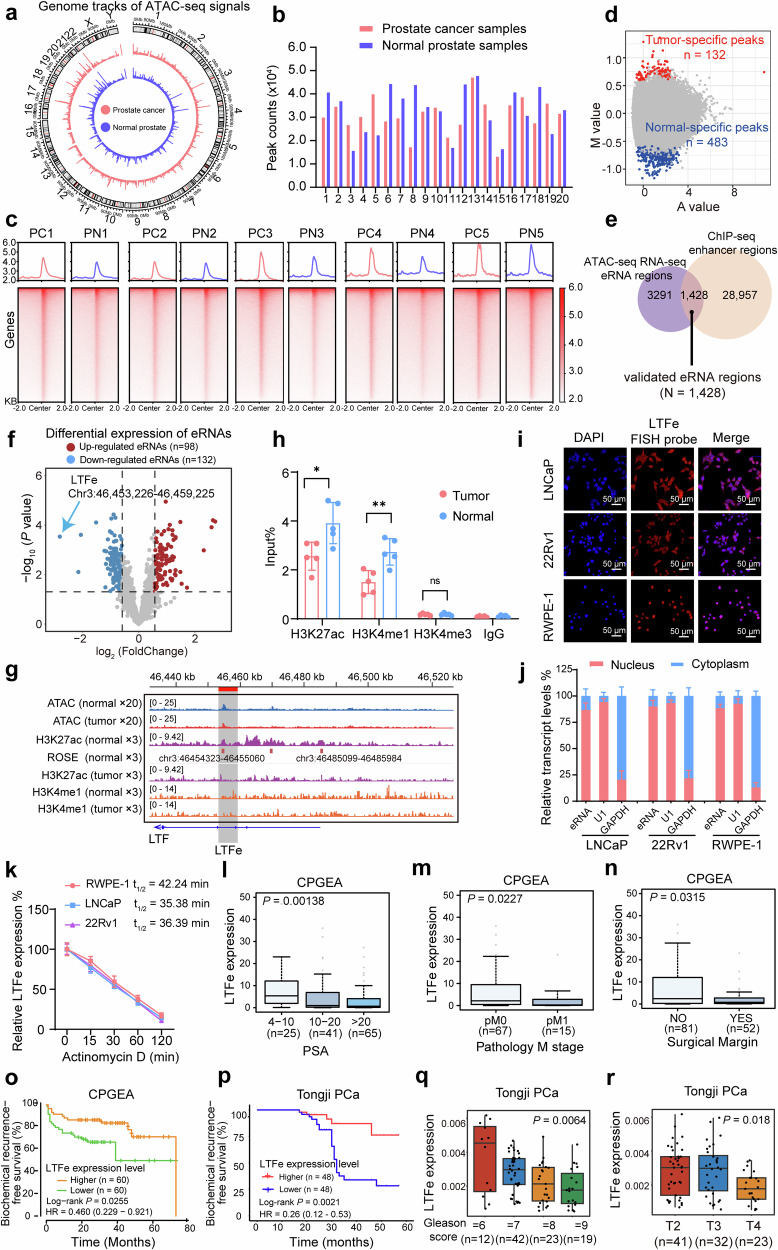
Chromatin accessibility and enhancer transcription profiling in human prostate cancer. **a** Genome-wide ATAC-seq signal tracks comparing chromatin accessibility between prostate cancer tissues and adjacent normal prostate tissues. Peaks indicate regions of open chromatin. **b** Distribution of ATAC-seq peaks identified across prostate cancer and normal prostate tissues, illustrating the extent of chromatin accessibility in each tissue type. **c** Representative heatmap showing the intensity of ATAC-seq signals within ± 2 Kb around the center of ATAC peaks in pairs of prostate cancer and normal prostate tissues. Signal intensities outside this region are not displayed, emphasizing the focal chromatin accessibility at these sites. **d** Tissue-specific peaks identified from normalized ATAC-seq data. The M value represents the log2 fold change, while the A value denotes the average log2 read count. Tumor-specific peaks are marked in red, and normal-specific peaks are indicated by blue. **e** Venn diagram illustrating the identification of 1428 enhancer RNA (eRNA) sites through integrated analysis of ATAC-seq, RNA-seq, and ChIP-seq data. **f** Volcano plot depicting the differential expression of annotated eRNAs. Red points represent significantly up-regulated eRNAs (fold changes > 1.5, *P* value < 0.05), while blue points represent significantly down-regulated eRNAs (fold changes < −1.5, *P* value < 0.05). Note that LTFe is the most downregulated eRNA in prostate tumors compared to the adjacent normal prostate tissue. **g** Epigenetic tracks from ATAC-seq in 20 pairs of prostate cancer and adjacent normal prostate tissues, combined with ChIP-seq data in from three prostate cancer and adjacent normal prostate tissue pairs. These tracks reveal the enrichment of active enhancer marks (ATAC-seq, H3K27ac, and H3K4me1) at the LTFe locus. **h** ChIP-qPCR results demonstrating differential enrichment of H3K27ac, H3K4me1, and H3K4me3 at the LTFe region in prostate cancer compared to normal prostate tissues. Data are presented as mean ± SD, with statistical significance assessed by a two-sided Student’s *t* test (**P < 0.01, *P < 0.05). **i** Confocal RNA fluorescence in situ hybridization images showing subcellular localization of LTFe in LNCaP, 22Rv1 and RWPE-1. The LTFe antisense probe is labeled in red, while nuclei are counterstained with DAPI (blue). **j** Subcellular localization of LTFe in three cell lines determined by real-time qPCR. U1 serves as a nuclear marker, and GAPDH serves as a cytoplasmic marker, indicating higher nuclear concentration of LTFe. **k** eRNA half-life assay following Actinomycin D treatment in RWPE-1, LNCaP, and 22Rv1 cells. Data are normalized to the LTFe expression at ‘0 min’**. l**, **m** The LTFe downregulation correlating with higher levels of PSA (**l**), tumor metastasis (**m**), and positive surgical margin (**n**) in a cohort of prostate cancer patients. **o**, **p** Kaplan–Meier survival analysis showing an increased risk of biochemical recurrence (BCR) in patients with lower LTFe expression levels in a public cohort (**o**) and our in-house prostate cancer cohort (**p**). Statistical significance was evaluated using the log-rank test. **q**, **r** In the Tongji cohort, higher LTFe expression is significantly associated with a lower Gleason score and less advanced T stage. Statistical significance was determined using the Kruskal–Wallis test

To delineate chromatin accessibility alterations in prostate tumorigenesis, we conducted ATAC-seq and RNA-seq analyses on twenty paired prostate cancer and adjacent normal prostate tissues. The genome-wide ATAC-seq signal tracks illustrated distinct chromatin accessibility patterns between cancerous and normal tissues, with a clear divergence in accessible regions (Fig. 1a). Correlation analysis of chromatin accessibility within each group displayed strong intra-group similarity, effectively differentiating cancer from normal samples (Supplementary Fig. 1a).

To identify accessible chromatin regions, we applied rigorous peak detection criteria (*P* < 0.05, peak length < 500 bp, and occurrence in at least five samples). This yielded between 16,012 and 44,673 peaks in prostate cancer tissues and 13,496 to 47,630 peaks in normal tissues (Fig. 1b). Genomic annotation revealed that most open chromatin regions were found in intronic and intergenic regions, suggesting these regions harbor potential enhancers (Supplementary Fig. 1b, c).^18^ Notably, a large proportion of ATAC-seq signals were concentrated within ±2 kb of peak centers, suggesting a defined chromatin accessibility structure (Fig. 1c, Supplementary Fig. 1d–f).

We further examined chromatin accessibility changes across individual samples to uncover biologically relevant patterns. Tissue-specific peaks were defined using differential analysis criteria (|fold change| > 1.5, *p*-value < 0.05), calculated with M (log2 fold change) and A (average log2 read count) values. This analysis identified 132 tumor-specific and 483 normal-specific peaks **(**Fig. 1d**)**, which effectively stratified samples into tumor and normal groups (Supplementary Fig. 1g). Notably, both groups exhibited significantly lower enrichment within 5 kb of transcription start sites (TSS) (Supplementary Fig. 1h, i). Functional annotation and pathway enrichment analysis using GREAT^19^ revealed critical biological processes, including protein-to-membrane docking and peroxisomal transport, providing insights into chromatin dynamics underlying cancer progression (Supplementary Fig. 1j).

Next, we explored the relationship between chromatin accessibility and gene expression during prostate cancer development. RNA-seq analysis identified 200 differentially expressed genes (DEGs), and ATAC-seq peaks associated with these DEGs were selected for further analysis. Spearman correlation analysis between normalized ATAC-seq signals and expression levels of neighboring genes identified peaks with strong correlations (*R* > 0.3, *p* < 0.05). This revealed significant associations between chromatin accessibility and gene expression, underscoring their interplay in prostate tumorigenesis (Supplementary Fig. 2a).

Focusing on enhancer regions, we excluded peaks within± 2.5 kb of TSS to minimize promoter contamination. This yielded 13,176 to 41,893 putative enhancers across the samples (Supplementary Fig. 2b). Integrating RNA-seq data refined this list to 4719 enhancer RNAs (eRNAs). To validate these findings, we incorporated chromatin immunoprecipitation sequencing (ChIP-seq) data for H3K27ac and H3K4me1 in three paired prostate cancer and adjacent normal prostate tissues, capturing a total of 30,385 enhancer regions. Among these, 1428 of the previously identified 4719 eRNAs were validated (Fig. 1e).

Differential expression analysis identified 98 eRNAs significantly upregulated and 132 downregulated in tumors (Fig. 1f, Data S1). Notably, over 45% of eRNAs exhibited strong positive correlation (*R* > 0.3) with the expression of neighboring genes in both normal (46.75%) and tumor samples (56.62%) (Supplementary Fig. 2c). Pathway enrichment analysis of these neighboring genes using Gene Ontology (GO), Kyoto Encyclopedia of Genes and Genomes (KEGG), WikiPathway, and Reactome revealed associations with processes such as cell differentiation, FoxO signaling, and programmed cell death (Supplementary Fig. 2d).

Given the pivotal role of RNA binding proteins (RBPs) in eRNA function,^20^ RBP enrichment analysis identified potential interactions between eRNAs and RBPs, with a notable emphasis on members of the heterogeneous nuclear ribonucleoprotein (HNRNP) families (Supplementary Fig. 2e).

Collectively, these results provide a comprehensive characterization of enhancer-driven transcription in prostate cancer, offering critical insights into its epigenomic landscape and highlighting potential regulatory mechanisms driving tumorigenesis.

### Enhancer transcriptional landscape analyses identify an eRNA associated with prostate cancer development

Our analysis of eRNA expression identified a notable candidate, an eRNA located at chr3:46,453,226-46,459,225, near the lactotransferrin (*LTF*) gene. This eRNA, hereafter referred to as *LTFe*, was most significantly downregulated in prostate cancer tissues compared to adjacent normal prostate tissues (Fig. 1f, Supplementary Fig. 2f).

To validate the presence and functional significance of *LTFe*, we employed a series of experimental approaches. Elevated ATAC-seq signals at the *LTFe* locus indicated increased chromatin accessibility, a characteristic of active regulatory regions (Fig. 1g). Supporting this, ChIP-seq data revealed that *LTF*e resided within an active enhancer region, with significantly higher H3K27ac/H3K4me1 signal enrichment in normal tissues compared to tumor tissues (Fig. 1g). To confirm these findings, we performed ChIP-qPCR assays on prostate cancer and adjacent normal tissue samples. The results were consistent with sequencing datasets, demonstrating significant enrichment of H3K27ac and H3K4me1 in normal prostate tissues, while these marks were markedly reduced in cancerous tissues, supporting the hypothesis that *LTFe* functions as an active enhancer suppressed in the cancer state (Fig. 1h).

Further investigation into the subcellular localization of *LTFe* was conducted using RNA fluorescence in situ hybridization (RNA-FISH) and subcellular fractionation assays in prostate cancer cell lines (LNCaP and 22Rv1) and the normal prostatic epithelial cell line (RWPE-1). Results indicated that *LTFe* predominantly localized to the nucleus (Fig. 1i, j), suggesting a regulatory role at the chromatin level, consistent with its characterization as an enhancer RNA. To comprehensively characterize LTFe, we assessed its half-life and stability in different cell lines using actinomycin D assays. LTFe exhibited a longer half-life in the normal prostatic epithelial cell line RWPE-1 (42.24 min) compared to the prostate cancer cell lines LNCaP (35.38 min) and 22Rv1 (36.39 min) (Fig. 1k). These findings suggest that LTFe stability is reduced in prostate cancer cells, potentially contributing to its dysregulation in the tumor context.

We next examined the clinical relevance of *LTFe* by assessing its expression in a cohort of 134 prostate cancer patients. The results showed that higher *LTFe* expression was significantly associated with lower serum PSA levels (Fig. 1l), reduced metastasis (Fig. 1m), and fewer positive surgical margins (Fig. 1n). Kaplan–Meier survival analysis further demonstrated that elevated *LTFe* expression was strongly associated with a reduced risk of biochemical recurrence (BCR) (Fig. 1o). Similar trends were observed in our in-house cohort of 96 prostate cancer patients, where higher *LTFe* levels correlated with favorable clinical outcomes, including reduced risk of BCR, lower Gleason scores and earlier T stages (Fig. 1p–r).

To gain a deeper understanding of LTFe, we attempted to predict both its secondary and tertiary structures. The secondary structure of LTFe was initially calculated using RNAfold^21^ (Supplementary Fig. 3a and Supplementary Table 1). Subsequently, the tertiary structure was predicted using 3dRNA.^22^ The top three predicted models, with respective prediction scores of 26.3773, 26.4581, and 26.4586, are shown in Supplementary Fig. 3b–d, providing valuable insights into the potential configuration of *LTF*.

Taken together, our results highlight *LTFe* as a potential regulator of prostate cancer development. Its suppression in cancer tissues likely contributes to the downregulation of critical enhancer-driven regulatory elements. The strong association between high *LTFe* expression and improved clinical outcomes, including reduced risk of BCR, suggests that *LTFe* could serve as a therapeutic target and a biomarker in prostate cancer.

### *LTFe* suppresses prostate cancer proliferation both in vitro and in vivo

**Fig. 2:**
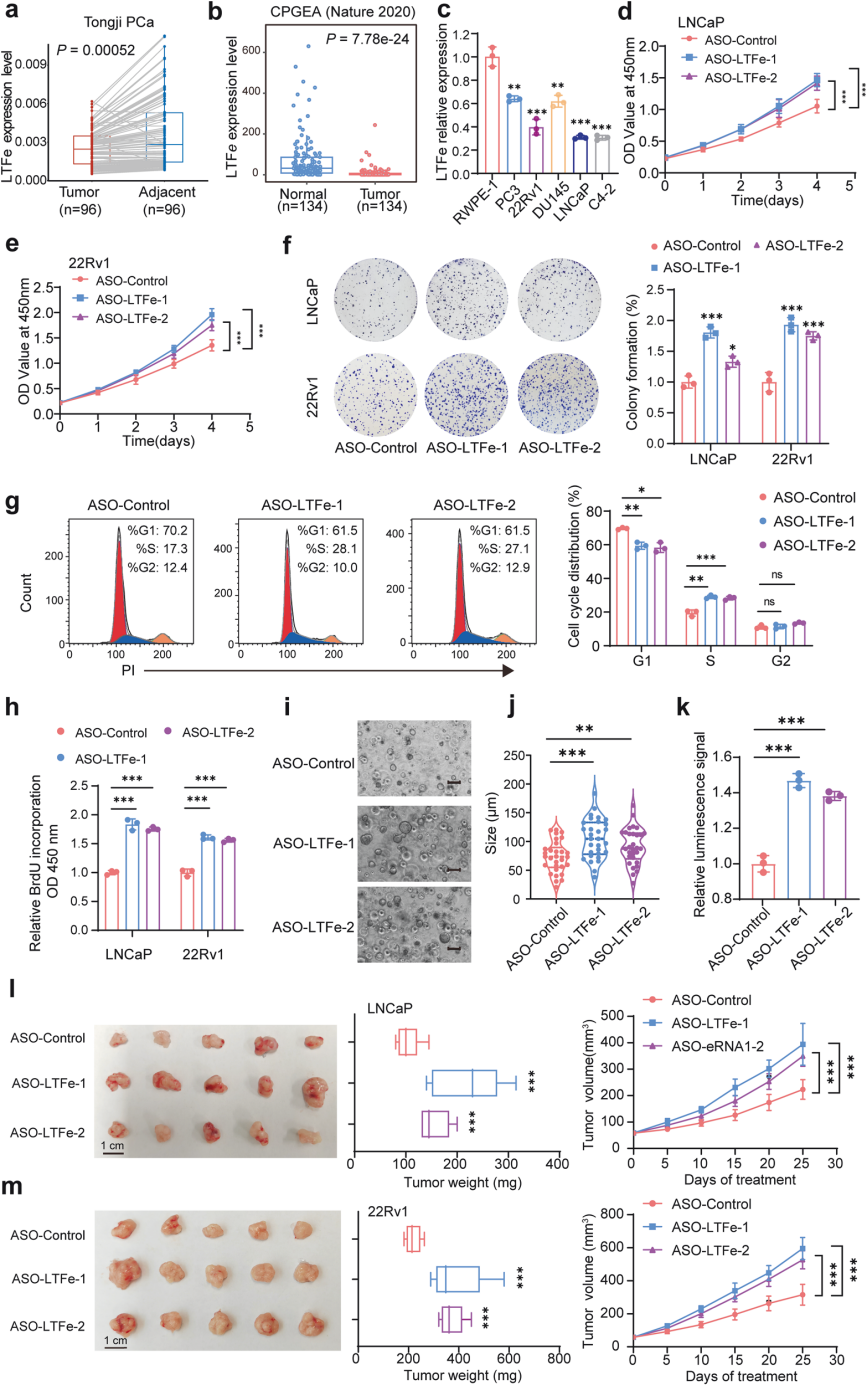
LTFe exhibits tumor-suppressive functions in prostate cancer. **a**, **b** Analysis of prostate cancer tissue samples from our in-house prostate cancer cohort (**a**) and a public cohort (**b**) revealed significantly lower LTFe expression in prostate tumor tissues compared to normal tissues. **c** Real-time qPCR analysis of LTFe expression levels (normalized to *GAPDH*) across various prostate cell lines, showing reduced expression in cancerous compared to normal prostatic epithelial cells. **d**, **e** CCK-8 assays demonstrating a significant increase in the proliferation of prostate cancer cells following LTFe knockdown. **f** Colony formation assays revealed a substantial increase in colony numbers in both LNCaP and 22Rv1 cells after LTFe knockdown, indicating enhanced tumorigenic potential. Data are shown as mean ± SD. **g** Flow cytometry analysis of the cell cycle profile in LNCaP cells after LTFe knockdown. **h** BrdU assay measuring the proliferation of prostate cancer cells following LTFe knockdown. **i** Representative patient-derived organoid images treated with ASO-LTFe for 14 days. Scale bar, 100 μm. **j** Quantification of organoid size (*n* = 10 fields from three representative experiments). **k** ATP levels (Luminescence signal) of organoid treated with ASO-LTFe were shown. **l**, **m** Tumor volume and weight measurements in xenografts derived from LNCaP and 22Rv1 cells treated with ASO-LTFe. Tumors received intratumoral injections of 10 nmol ASO when they reached approximately 50–100 mm³, with injections administered every three days for a total of eight treatments. Tumor volumes were recorded every five days. *P < 0.05, **P < 0.01, ***P < 0.001. Data are presented as mean ± SD, *n* = 3 biologically independent experiments for panels (**c**)–(**h**); *n* = 5 mice per group for panels (**l**), (**m**). Statistical analyses are performed using a two-sided Student’s *t* test for panels (**b**), (**c**), (**f**)–(**k**), tow-way ANOVA in (**d**), (**e**), (**l**), (**m**), and a paired sample *t* test for panel (**a**)

To elucidate the functional role of *LTFe* in prostate cancer, we first examined its expression levels in tumor tissues compared to adjacent normal prostate tissues using our own prostate cancer tissue cohort. This analysis revealed significantly lower *LTFe* expression in tumor tissues relative to normal tissues (Fig. 2a). The finding was corroborated in a publicly available Chinese prostate cancer cohort,^5^ which similarly exhibited markedly lower *LTFe* expression in tumors (Fig. 2b). Interestingly, when we analyzed the TCGA PRAD cohort, which consists predominantly of patients of European descent, we did not observe the same differential expression pattern (Supplementary Fig. 4a). This discrepancy suggests a potential population-specific variation in *LTFe* regulation, which may reflect underlying genetic and epigenetic differences in prostate cancer across ethnic groups.

Consistent with our tissue-based findings, LTFe expression was also universally downregulated across multiple prostate cancer cell lines (PC3, 22Rv1, DU145, LNCaP, and C4-2) compared to the non-malignant RWPE-1 prostate epithelial cell line (Fig. 2c). These observations collectively indicate that LTFe downregulation is a recurrent event in prostate cancer and may play a fundamental role in disease progression.

To investigate the impact of *LTFe* downregulation on prostate cancer cell behavior, we designed two antisense oligonucleotides (ASOs) specifically targeting *LTFe*. Quantitative PCR (qPCR) confirmed the effective knockdown of *LTFe* by both ASOs (Supplementary Fig. 4b). Functional assays were then conducted to assess the biological consequences of *LTFe* depletion. The Cell Counting Kit-8 (CCK-8) assay demonstrated a significant increase in prostate cancer cell proliferation following *LTFe* knockdown (Fig. 2d, e). Similarly, colony formation assays revealed a marked increase in the number of colonies formed by LTFe-deficient cells (Fig. 2f). To further elucidate the impact of *LTF*e on prostate cancer cell proliferation, we examined the effects of *LTF*e knockdown on cell cycle progression and BrdU incorporation. Knockdown of *LTF*e accelerated the progression of the cell cycle into the S phase (Fig. 2g, Supplementary Fig. 4c), and BrdU assays confirmed enhanced proliferation capacity of cancer cells upon LTFe depletion (Fig. 2h). Moreover, we performed an organoid assay to explore the autonomous role of *LTFe*. Consistent with the observed phenotype in cancer cells, the treatment of ASO-*LTFe* markedly enhanced the ability of tumor to form organoids, along with a notable increase in cell proliferation (Fig. 2i–k).

We extended our investigation to an in vivo context using a xenograft model. Mice were injected with prostate cancer cells treated with ASO-*LTFe*, and the results showed that *LTFe* knockdown significantly accelerated tumor growth. Tumors in the ASO-*LTFe* treated groups were notably larger and heavier compared to those in the control group (Fig. 2l, m). These findings strongly suggest that *LTFe* functions as a tumor suppressor in prostate cancer, inhibiting both cell proliferation and tumor growth.

To further validate the tumor-suppressive function of *LTFe*, we performed overexpression studies in prostate cancer cell lines. The overexpression of *LTFe* resulted in a significant reduction in both proliferation and colony formation abilities of prostate cancer cells in vitro (Supplementary Fig. 4d–g). In vivo, subcutaneous xenografts in mice established from LTFe overexpressing cells exhibited significantly slower growth rates compared to control xenografts (Supplementary Fig. 4h, i), corroborating the tumor-suppressive effects observed in vitro.

Collectively, these in vitro and in vivo experiments consistently demonstrate that *LTFe* acts as a potent inhibitor of prostate cancer proliferation and tumor growth. The use of both knockdown and overexpression approaches provides compelling evidence for the tumor-suppressive role of *LTFe* in prostate cancer. These findings highlight *LTFe* as a potential therapeutic target for prostate cancer treatment.

### LTFe upregulates LTF expression in prostate cancer

**Fig. 3:**
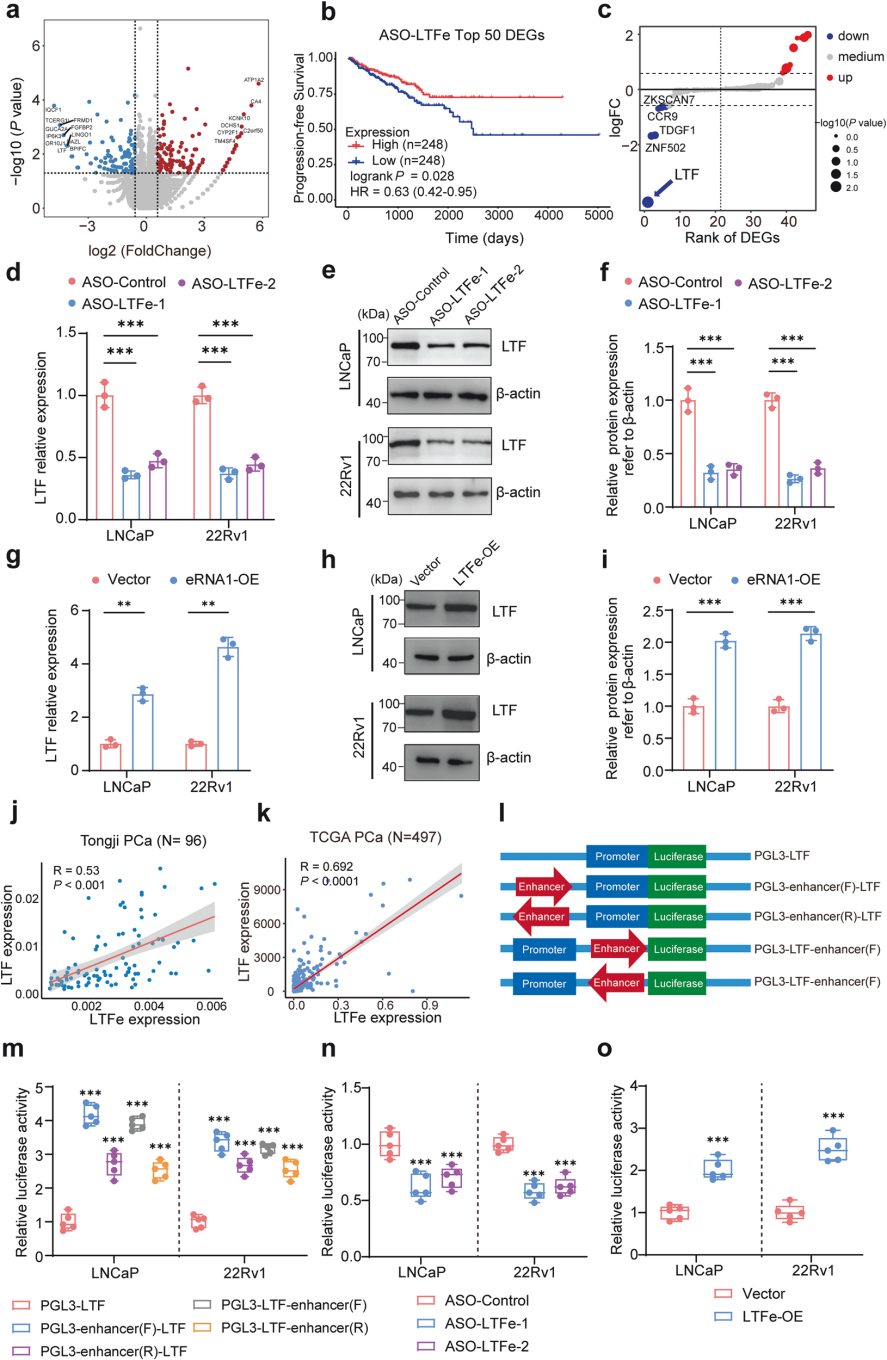
LTFe promotes LTF expression in prostate cancer. **a** Volcano plot illustrating differential gene expression from RNA-seq analysis in LNCaP cells. Genes with fold changes > 1.5 and *P*-value < 0.05 are highlighted. Red points represent genes with high expression in ASO-Control cells, while blue points denote lower expression. **b** Kaplan–Meier survival analysis showing that reduced expression of the top 50 LTFe-associated genes correlates with better clinical outcomes in prostate cancer patients. **c** Expression analysis of 46 genes located within a 1MB range of the LTFe center. Blue points indicate decreased expression, red points indicate increased expression following LTFe knockdown. **d** Real-time qPCR assays showing that RNA levels of *LTF* are significantly modulated in response to LTFe knockdown. **e**, **f** Western blot quantification revealing alterations in LTF protein levels following LTFe knockdown. **g**–**i** Real-time qPCR and Western blot assays demonstrating that both RNA and protein levels of LTF are modulated upon LTFe overexpression. **j**, **k** Correlation analysis of LTFe and *LTF* expression levels in our patient cohort (**j**) and the TCGA cohort (**k**). Pearson correlation coefficients (*R*) and corresponding *P* values are provided. **l** Schematic diagram of plasmids used in dual-luciferase reporter assays to assess the impact of LTFe on *LTF* promoter activity. **m** Dual-luciferase assays reveal that the chromatin region associated with LTFe enhances *LTF* promoter activity, independent of the orientation or sequence arrangement of the enhancer region. **n**, **o** Dual-luciferase assays showing that LTFe knockdown significantly reduces, whereas its overexpression increases, *LTF* promoter activity. *P < 0.05, **P < 0.01, ***P < 0.001. Data are presented as mean ± SD from *n* = 3 biologically independent experiments in (**d**), (**f**), (**g**), (**i**), and *n* = 5 in (**m**)–(**o**). Statistical significance was determined using a two-sided Student’s *t* test for (**d**), (**f**), (**g**), (**i**), (**m**), (**n**) and (**o**)

Given the emerging role of eRNAs in modulating gene expression implicated for oncogenesis and tumor proliferation,^23,24^ we sought to identify potential downstream targets of *LTFe* to better understand its impact on prostate cancer advancement. To achieve this, we performed RNA-seq analysis on prostate cancer cell lines treated with ASOs targeting *LTFe*. Our analysis identified 149 genes with decreased expression and 147 genes with increased expression following *LTFe* knockdown (Fig. 3a).

To assess the clinical relevance of these DEGs, we analyzed their association with survival outcomes in prostate cancer patients. Kaplan–Meier analysis revealed that lower expression of the top 50 *LTFe*-associated genes correlated with better clinical outcomes (Fig. 3b). Considering that eRNAs typically regulate genes within the same topologically associated domain (TAD) through cis-interactions,^25,26^ we focused on the expression changes of 46 genes within a 1MB range of *LTFe*. Among these, lactotransferrin (*LTF*) emerged as the most significantly affected gene (Fig. 3c).

To validate this observation, we measured *LTF* expression levels in prostate cancer cell lines after *LTFe* knockdown or overexpression. As anticipated, both the RNA and protein levels of *LTF* were modulated in line with changes in *LTFe* expression (Fig. 3d–i). Additionally, analysis of prostate cancer tissue samples revealed a positive correlation between *LTFe* and *LTF* expression levels in both the Tongji and TCGA cohorts (Fig. 3j, k).

Further investigation using luciferase reporter assays confirmed that the chromatin region associated with *LTFe* significantly enhanced *LTF* promoter activity, independent of orientation or sequence order (Fig. 3l, m). Notably, *LTFe* knockdown markedly reduced *LTF* promoter activity, while *LTFe* overexpression significantly boosted promoter activity (Fig. 3n, o).

These results establish *LTFe* as a crucial enhancer RNA that upregulates *LTF* expression, underscoring its role in modulating gene expression critical to prostate cancer proliferation. This *LTFe*-*LTF* regulatory axis highlights the importance of enhancer RNAs in driving oncogenic processes and presents a potential therapeutic target for managing prostate cancer.

### LTFe recruits HNRNPF to enhance LTF transcription through chromatin loop formation

Emerging evidence suggests that eRNAs often do not function in isolation but instead interact with RBPs to form complexes that play critical roles in regulating gene transcription.^23,27^ Based on this, we hypothesized that *LTF*e may exert its effects on *LTF* transcription through interactions with specific RBPs. To test this hypothesis, we employed a chromatin isolation by RNA purification (ChIRP) assay to capture and isolate both the RBPs and genomic regions associated with *LTF*e in prostate cancer cells.

**Fig. 4:**
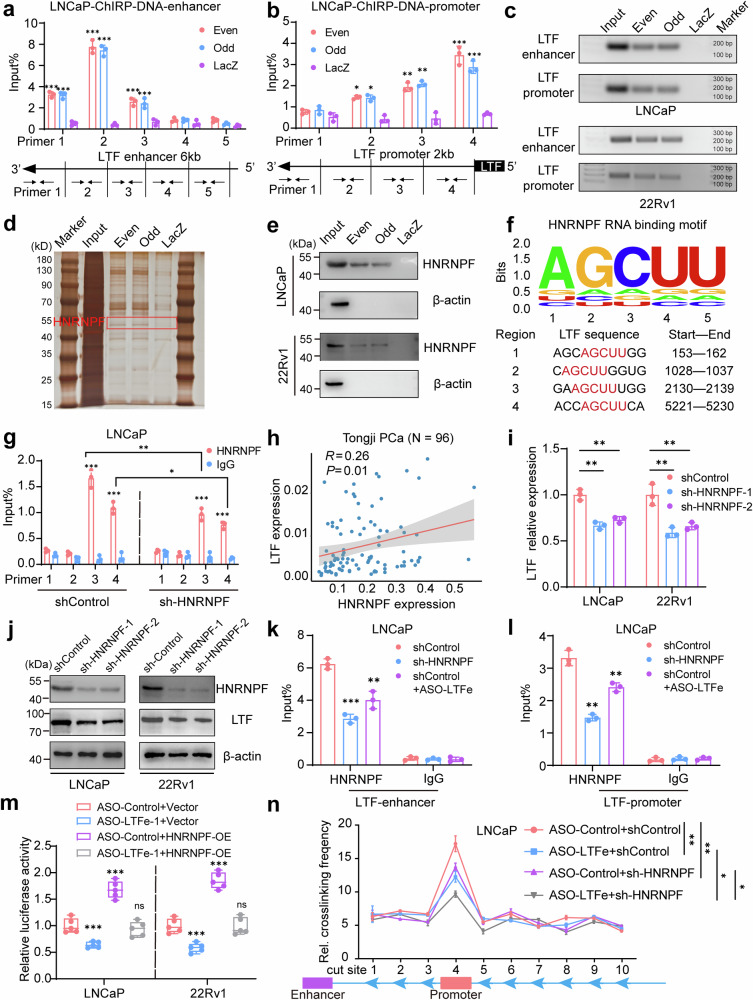
LTFe recruits HNRNPF to enhance LTF transcription via chromatin loop formation. **a**, **b** Chromatin isolation by RNA purification (ChIRP) assays were performed in LNCaP and 22Rv1 cells using two sets of antisense probes targeting *LTF*e (“even group” and “odd group”) and control probes (anti-LacZ). Genomic DNA enrichment was quantified by qRT-PCR using primers spanning a 6 kb region of the *LTF* enhancer and primers targeting the *LTF* promoter. **c** Agarose gel electrophoresis results showing genomic DNA enrichment from the ChIRP assays. **d** Silver staining results illustrating protein complexes binding to the *LTF* enhancer region in LNCaP cells. **e** Western blot analysis confirming HNRNPF as a candidate partner of *LTF*e. **f** Identification of four potential *LTF*e-HNRNPF binding sites through RBP motif analysis, utilizing data from the StarBase database. **g** RIP-qPCR experiments revealed significant binding of HNRNPF to *LTF*e region 3 in LNCaP cells, with decreased binding observed following HNRNPF knockdown. **h** Pearson correlation analysis demonstrating a positive correlation between HNRNPF and *LTF* expression levels in the Tongji prostate cancer cohort. **i** qRT-PCR assay showing changes in *LTF* transcript levels (normalized to *GAPDH*) in LNCaP and 22Rv1 cells transfected with shControl or sh-HNRNPF. **j** Western blot analysis displaying *LTF* protein levels in LNCaP and 22Rv1 cells transfected with shControl or sh-HNRNPF. **k**, **l** ChIP-qPCR assays confirming HNRNPF binding to the *LTF* enhancer (primer 2) and promoter (primer 4) regions in LNCaP cells. The binding intensity varied with the expression levels of HNRNPF and *LTF*e. **m** Dual-luciferase assays showing the activity of the enhancer-*LTF* promoter reporter (pGL3-enhancer-*LTF*) in LNCaP and 22Rv1 cells stably transfected with ASO-Control, ASO-*LTF*e, or co-transfected with an empty vector or HNRNPF overexpression (HNRNPF-OE) plasmid. **n** Quantification of 3C profiles in LNCaP cells transfected with ASO-Control, ASO-*LTF*e, sh-HNRNPF, or a combination of ASO-*LTF*e and sh-HNRNPF. Interaction frequencies between the *LTF* enhancer (anchor) and ten Sacl enzyme cutting sites, including the *LTF* promoter region, are shown in a dot plot. *P < 0.05, **P < 0.01, ***P < 0.001. Data are presented as mean ± SD from *n* = 3 biologically independent experiments in (**a**), (**b**), (**g**), (**i**), (**k**), (**l**), (**n**), or *n* = 5 in (**m**). Statistical analysis is performed using a two-sided Student’s *t* test for (**a**), (**b**), (**g**), (**i**), (**k**), (**l**), (**m**), and one-way ANOVA for (**n**)

We first validated the specificity of our ChIRP assay by utilizing biotinylated antisense probes targeting LTFe, divided into “Odd” and “Even” groups. Both probes successfully enriched LTFe while excluding the GAPDH transcript, confirming the assay’s specificity (Supplementary Fig. 5a, b). Subsequent analysis identified a significant enrichment of DNA fragments corresponding to the LTF enhancer (chr3:46,454,426-46,455,625) and promoter regions (chr3:46,485,234-46,485,734), suggesting that LTFe directly interacts with these genomic regions to regulate LTF expression (Fig. 4a–c, Supplementary Fig. 5c, d).

To identify the protein partners involved in this regulatory mechanism, we analyzed the proteins enriched by the *LTFe* probes. Silver staining revealed distinct protein bands between 40 kDa and 55 kDa in the probe group compared to the control (Fig. 4d, Supplementary Fig. 5e). Mass spectrometry analysis pinpointed heterogeneous nuclear ribonucleoprotein F (HNRNPF), a 46 kDa protein, as being specifically enriched in the *LTFe*-associated fraction (Supplementary Fig. 5f). This interaction between *LTF*e and HNRNPF was further validated by western blotting, confirming HNRNPF enrichment in the probe group but not in the control (Fig. 4e).

Building on the discovery of *LTF*e’s interaction with HNRNPF, we aimed to identify the exact binding sites involved in this interaction. Using RBP motif analysis from the StarBase database,^28^ we identified four potential binding sites on *LTF*e where it could interact with HNRNPF (Fig. 4f). To experimentally validate these predictions, we conducted RNA immunoprecipitation followed by quantitative PCR (RIP-qPCR). Our results demonstrated that HNRNPF binding was particularly strong at *LTF*e region 3. Notably, this binding was significantly reduced upon HNRNPF knockdown (Fig. 4g, Supplementary Fig. 5g, h), confirming the specificity of the interaction. Furthermore, we observed a positive correlation between the expression levels of *HNRNPF* and *LTF* (Fig. 4h, Supplementary Fig. 5i), suggesting that HNRNPF could directly modulate *LTF* expression through its interaction with *LTF*e. Notably, like *LTF*, HNRNPF exhibited low expression levels in cancer (Supplementary Fig. 5j), further highlighting a potential regulatory mechanism within the tumor context.

To further test this hypothesis, we performed qRT-PCR and western blot analyses to evaluate *LTF* expression following stable knockdown of HNRNPF. Both *LTF* RNA and protein levels were significantly reduced after HNRNPF knockdown (Fig. 4i, j, Supplementary Fig. 5k), reinforcing the notion that HNRNPF is a crucial regulator of *LTF* expression. Furthermore, we found that HNRNPF could bind to specific regions within the *LTF* enhancer (primer 2) and promoter (primer 4), with binding intensity modulated by the expression levels of both HNRNPF and *LTF*e (Fig. 4k, l, Supplementary Fig. 5l, m). This suggests a synergistic effect between the two molecules in regulating *LTF* transcription. To directly test this synergy, we conducted qRT-PCR and dual luciferase reporter assays, which demonstrated that HNRNPF overexpression could reverse the reduction in *LTF* expression and promoter activity caused by *LTFe* knockdown (Fig. 4m, Supplementary Fig. 5n). These results further support the cooperative role of HNRNPF and *LTFe* in enhancing *LTF* transcription.

To investigate direct chromatin interactions between the *LTF* promoter and its enhancer, we performed chromosome conformation capture (3C) assays in LNCaP and 22Rv1 cells. The data provided compelling evidence of a robust physical interaction between the *LTF* enhancer and promoter, with significantly higher interaction frequencies at these sites compared to adjacent genomic regions (Fig. 4n, Supplementary Fig. 5o). These results indicate that the identified enhancer is actively engaging with the promoter to regulate *LTF* transcription. Notably, the knockdown of either *LTF*e or HNRNPF substantially reduced the frequency of these enhancer-promoter interactions, underscoring the critical role of these factors in maintaining chromatin loop integrity. The most pronounced reduction in interaction frequency occurred when both *LTF*e and HNRNPF were simultaneously knocked down, suggesting a synergistic role in facilitating the chromatin looping necessary for *LTF* transcription (Fig. 4n, Supplementary Fig. 5o).

To further investigate the interaction between HNRNPF and *LTF*e, we assessed the binding capability of HNRNPF to the predicted three-dimensional structure of *LTF*e. Using HDOCK, we calculated the binding efficiency between the two molecules. As anticipated, all three top-ranked *LTF*e structures successfully docked with HNRNPF, with docking scores exceeding an absolute value of 300 (Supplementary Fig. 3b–d). These results provide further support the ability of HNRNPF to bind with *LTF*e in the context of prostate cancer.

Taken together, these findings demonstrate that *LTF*e and HNRNPF collaborate to regulate *LTF* transcription by enhancing chromatin loop formation between key genomic regions. This interplay between *LTF*e and HNRNPF highlights the pivotal roles of eRNA and RBPs in modulating gene expression through chromatin architecture, providing important insights into the molecular mechanisms driving prostate cancer progression.

### LTFe suppresses prostate cancer proliferation through LTF activation

Our findings suggest that *LTFe* exerts tumor-suppressive effects by modulating chromatin architecture, leading to the transcriptional activation of *LTF*, which in turn inhibits prostate cancer proliferation in both in vitro and in vivo models. To further elucidate this mechanism, we hypothesized that the anti-tumor effects of *LTF*e are primarily mediated through the upregulation of *LTF* expression.

**Fig. 5:**
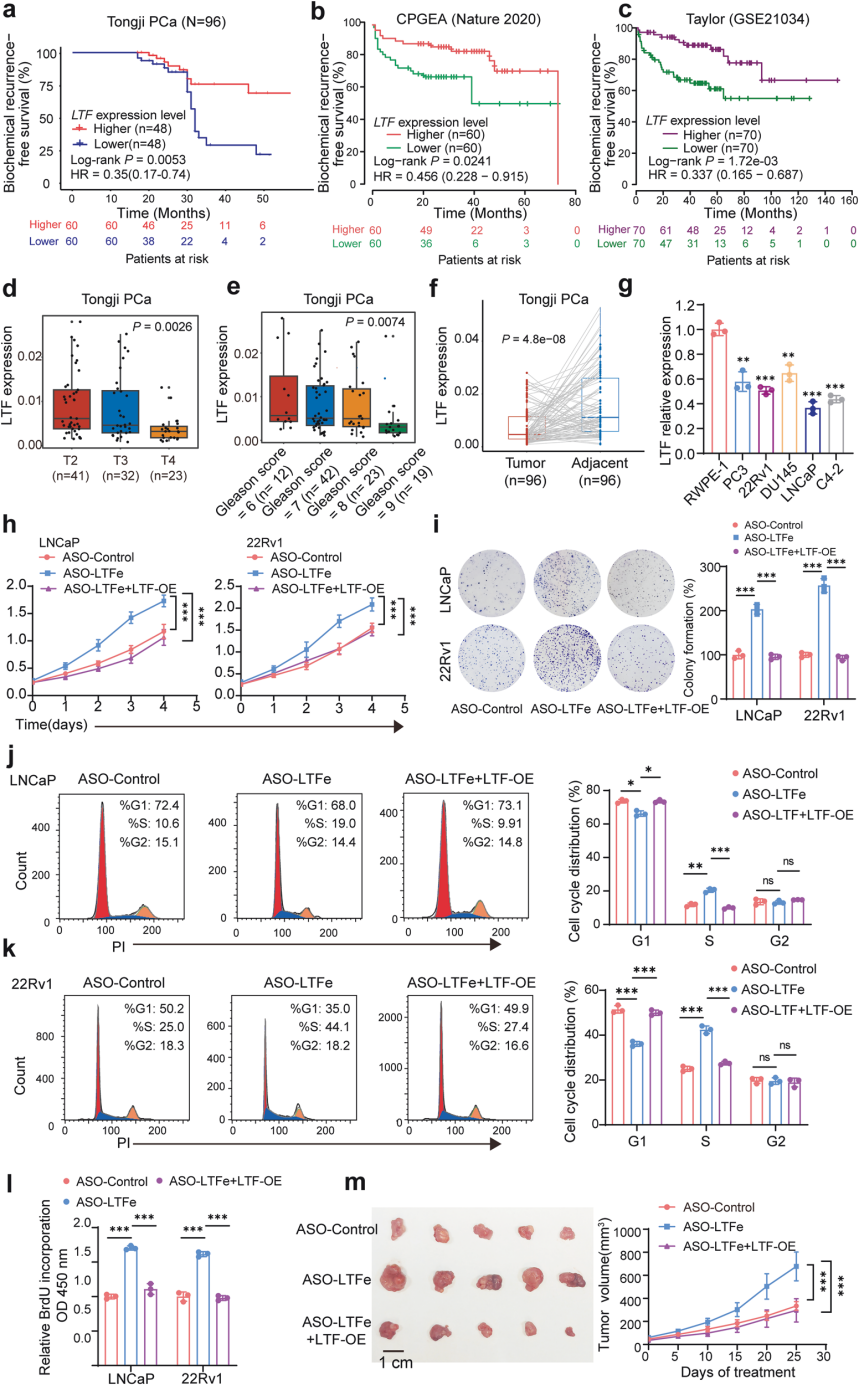
LTFe inhibits prostate cancer proliferation by targeting LTF. **a**–**c** Kaplan–Meier analysis showing that high *LTF* expression is significantly correlated with a reduced risk of biochemical recurrence (BCR) in prostate cancer patients from the Tongji cohort (**a**), and additionally two independent cohorts (**b**, **c**). **d**, **e** Higher *LTF* expression is associated with less advanced T stages and lower Gleason scores within the Tongji cohort. **f** *LTF* expression is significantly lower in tumor tissues compared to normal prostate tissues, as observed in our in-house prostate cancer tissue samples. **g** qRT-PCR analysis showing the relative expression of LTFe (normalized to *GAPDH*) across different prostate cell lines. **h** CCK-8 assays illustrating that the proliferation activation induced by ASO-*LTF*e transfection can be reversed by *LTF* overexpression. **i** Colony formation assays demonstrating that the enhanced colony formation due to ASO-*LTF*e transfection is reversible by *LTF* overexpression. **j**, **k** Flow cytometry analysis of the cell cycle in LNCaP (**j**) and 22Rv1 (**k**) cells showing that cell cycle alterations induced by ASO-LTFe transfection are reversible upon LTF overexpression. **l** BrdU assays confirming that the proliferation activation triggered by ASO-LTFe transfection is effectively reversed by LTF overexpression. **m** Overexpression of *LTF* counteracts the accelerated tumor growth rate induced by ASO-*LTF*e treatment in vivo. *P < 0.05, **P < 0.01, ***P < 0.001. Data are presented as mean ± SD, *n* = 3 biologically independent experiments in (**g**), (**h**), (**i**), (**j**), (**k**), (**l**) or *n* = 5 in (**m**). Statistical analysis is performed using a two-sided Student’s *t* test for (**g**), (**i**), (**j**), (**j**), (**k**), (**l**), Kruskal–Wallis test for (**d**), (**e**), tow-way ANOVA in (**h**), (**m**), log-rank test for (**a**–**c**), and paired sample *t* test for (**f**)

To test this, we first analyzed the impact of *LTF* expression on prostate cancer proliferation. Data from both public datasets and our in-house cohort revealed a strong association between high *LTF* expression and reduced risk of BCR in prostate cancer patients (Fig. 5a–c). Conversely, low *LTF* expression was significantly correlated with aggressive disease characteristics, such as higher Gleason scores and advanced T stages (Fig. 5d, e, Supplementary Fig. 6a, b). Additionally, *LTF* expression was markedly lower in prostate cancer tissues compared to adjacent non-cancerous tissues (Fig. 5f, Supplementary Fig. 6c). This observation was further corroborated by the finding that *LTF* levels in the non-cancerous prostatic epithelial cell line RWPE-1 were notably higher than those in various prostate cancer cell lines (Fig. 5g). These results align closely with the expression patterns observed in our *LTF*e analysis.

Next, to explore *LTF’s* role in prostate cancer, we generated *LTF*-overexpressing cell m. *LTF* overexpression significantly reduced cancer cell proliferation, as shown in Supplementary Fig. 6d. Similar to *LTF*e, elevated *LTF* expression exerted multiple inhibitory effects on prostate cancer proliferation. Specifically, overexpressed *LTF* reduced cell proliferation (Supplementary Fig. 6e), diminished colony formation ability (Supplementary Fig. 6f, g), induced G1 phase cell cycle arrest (Supplementary Fig. 6h, i), reduced BrdU uptake (Supplementary Fig. 6j), and suppressed tumorigenesis in vivo (Supplementary Fig. 6k). These findings highlight *LTF*’s potential role as a key suppressor of prostate cancer development.

To further assess the cis-regulatory relationship between *LTF*e on *LTF* during prostate cancer proliferation, we conducted rescue experiments in LNCaP and 22Rv1 cells. Knockdown of *LTF*e resulted in significant pro-tumorigenic changes, including enhanced cell proliferation (Fig. 5h), increased colony formation (Fig. 5i), a shift in the cell cycle to the S phase (Fig. 5j, k), elevated BrdU uptake (Fig. 5l), and accelerated tumor growth in vivo (Fig. 5m). Importantly, these pro-tumorigenic effects were fully reversed by LTF overexpression, emphasizing the regulatory interplay between LTFe and LTF (Fig. 5h–m). Moreover, stable knockdown of *LTF* significantly promoted cell proliferation and enhanced colony formation (Supplementary Fig. 6m, n), while also reversing the growth suppression induced by *LTF*e overexpression (Supplementary Fig. 6o, p).

Together, these results provide compelling evidence that *LTF* plays a pivotal role in suppressing prostate cancer proliferation. The tumor-suppressive effects of *LTF*e are primarily mediated through *LTF* activation, underscoring the therapeutic potential of targeting the *LTF*e-*LTF* axis in prostate cancer treatment.

### LTFe-LTF axis promotes ferroptosis through iron transport

**Fig. 6:**
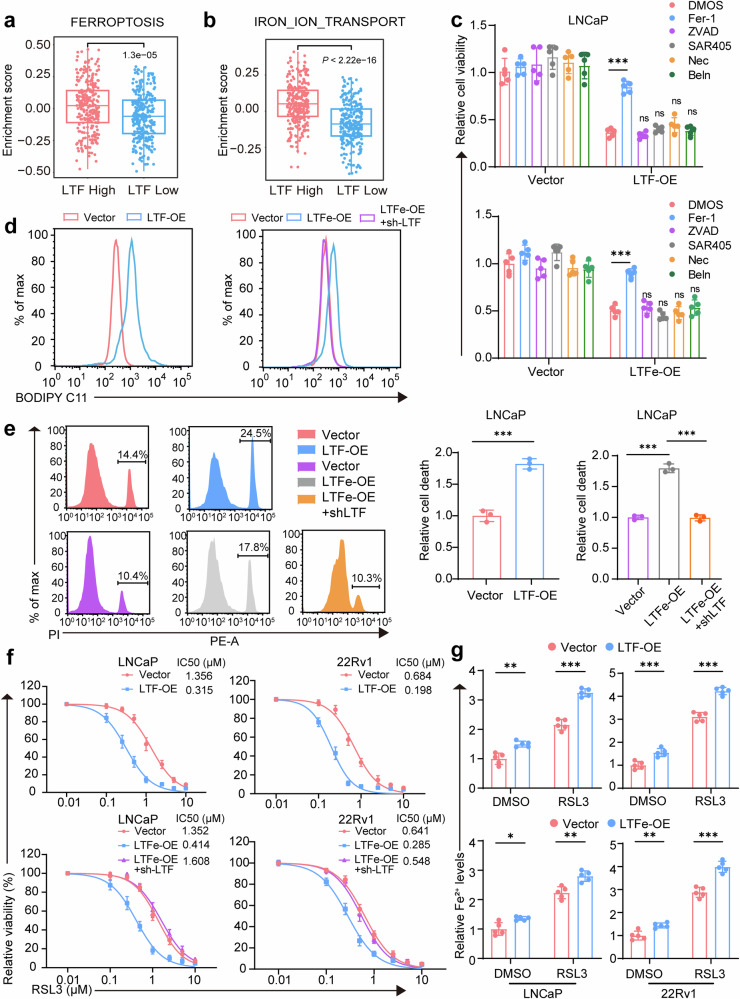
LTFe-LTF axis promotes ferroptosis in prostate cancer. **a**, **b** GSVA pathway enrichment box plots illustrating the enrichment of FERROPTOSIS and IRON_ION_TRANSPORT pathways in the TCGA prostate cancer cohort, highlighting the role of *LTF* in these processes. **c** Cell viability assay in LNCaP cells transfected with vector, *LTF*-OE or *LTF*e-OE, with and without pretreatment using the ferroptosis inhibitor ferrostatin-1 (Fer-1, 10 μM), the apoptosis inhibitor Z-VAD-FMK (ZVAD, 25 μM), the autophagy inhibitor SAR405 (1 μM), the necroptosis inhibitor necrostatin-1 (Nec, 25 μM), or the pyroptosis inhibitor belnacasan (Beln, 25 μM). **d** Lipid Reactive Oxygen Species (LipROS) levels in LNCaP cells transfected with vector, *LTF*-OE, *LTF*e-OE, or *LTF*e-OE+sh-*LTF*, measured using C11-BODIPY fluorescence, indicating oxidative stress related to ferroptosis. **e** Cell death rates in LNCaP cells transfected with vector, *LTF*-OE, *LTF*e-OE, or *LTF*e-OE+sh-*LTF*, determined by flow cytometry, illustrating the impact of *LTF* and *LTF*e on ferroptosis. **f** 24-h dose–response curves for RSL3 treatment in LNCaP and 22Rv1 cells transfected with vector, *LTF*-OE, *LTF*e-OE, or *LTF*e-OE+sh-*LTF*, demonstrating enhanced sensitivity to ferroptosis upon *LTF* and *LTF*e overexpression. **g** Changes in Fe^2+^ levels in LNCaP and 22Rv1 cells pretreated with DMSO or 1 μM RSL3 for 24 h, highlighting the role of *LTF*e-*LTF* in iron metabolism related to ferroptosis. *P < 0.05, **P < 0.01, ***P < 0.001. Data are presented as mean ± SD, *n* = 3 biologically independent experiments in (**e**) or *n* = 5 in (**c**) (**f**) (**g**). Statistical analysis is performed using two-sided Student’s *t* test

*LTF* is well-established in regulating iron metabolism and has been increasingly linked to ferroptosis, a form of programmed cell death driven by iron-dependent lipid peroxidation.^29–31^ Given this connection, we investigated the role of *LTF* in prostate cancer progression through ferroptosis. Gene set variation analysis (GSVA) revealed a strong association between *LTF* expression and iron-related pathways, particularly “FERROPTOSIS” and “IRON_ION_TRANSPORT” (Fig. 6a, b). High *LTF* expression corresponded with increased pathway enrichment scores, suggesting a key role for LTF in promoting ferroptosis in prostate cancer cells.

To assess whether the reduction in prostate cancer cell viability following *LTF* or *LTF*e overexpression was mediated by ferroptosis, we utilized inhibitors of various cell death pathways. Only the ferroptosis inhibitor ferrostatin-1 (Fer-1) was able to restore cell viability, which had been diminished by *LTF* or *LTF*e overexpression (Fig. 6c, Supplementary Fig. 7a, b). Other inhibitors targeting apoptosis (Z-VAD-fmk), autophagy (SAR405), necroptosis (Necro-1), and caspase-1 (Belnacasan) had no effect. These results indicate that *LTF* or LTFe-induced reduction in cell viability is specifically mediated through ferroptosis, ruling out other programmed cell death pathways.

Next, we examined the impact of *LTF*e on key ferroptosis markers. Overexpression of *LTF* resulted in a significant increase in lipid reactive oxygen species (LipROS), a hallmark of ferroptosis, in prostate cancer cells. This increase in LipROS due to *LTF*e overexpression was reversed by *LTF* knockdown (Fig. 6d, Supplementary Fig. 7c), confirming that *LTF* is a key mediator of *LTF*e-induced ferroptosis. Similarly, *LTF*e overexpression-induced cell death was mitigated by *LTF* reduction (Fig. 6e, Supplementary Fig. 7d), further supporting the critical role of LTF in mediating ferroptosis triggered by *LTFe*.

We then explored whether the *LTF*e-*LTF* axis could enhance the sensitivity of prostate cancer cells to ferroptosis-inducing agents. Treatment with RSL3, a selective ferroptosis inducer,^32^ significantly increased cell death in cells overexpressing either *LTF* or *LTF*e (Fig. 6f). This was accompanied by elevated levels of malondialdehyde (MDA), a key indicator of lipid peroxidation, further validating ferroptosis as the mechanism of action (Supplementary Fig. 7e, f). Additionally, overexpression of *LTF*e or *LTF* increased intracellular Fe^2+^ levels, which were further amplified by RSL3 treatment (Fig. 6g).

Collectively, our findings indicate that the *LTF*e-*LTF* axis promotes ferroptosis in prostate cancer cells by regulating iron transport and enhancing sensitivity to ferroptosis-inducing agents like RSL3. Targeting this axis represents a promising therapeutic strategy to induce ferroptosis and combat prostate cancer advancement, opening new avenues for effective treatment.

### AR signaling suppresses ferroptosis by regulating the LTFe-LTF axis

Given the critical role of AR signaling in prostate cancer progression through the modulation of genes essential for cellular proliferation and survival, we investigated whether AR influences ferroptosis by regulating the *LTFe-LTF* axis. Since ferroptosis is driven by iron-dependent lipid peroxidation, we hypothesized that AR could suppress this process by downregulating *LTFe* and its downstream target *LTF*, thereby promoting cancer cell survival.

**Fig. 7:**
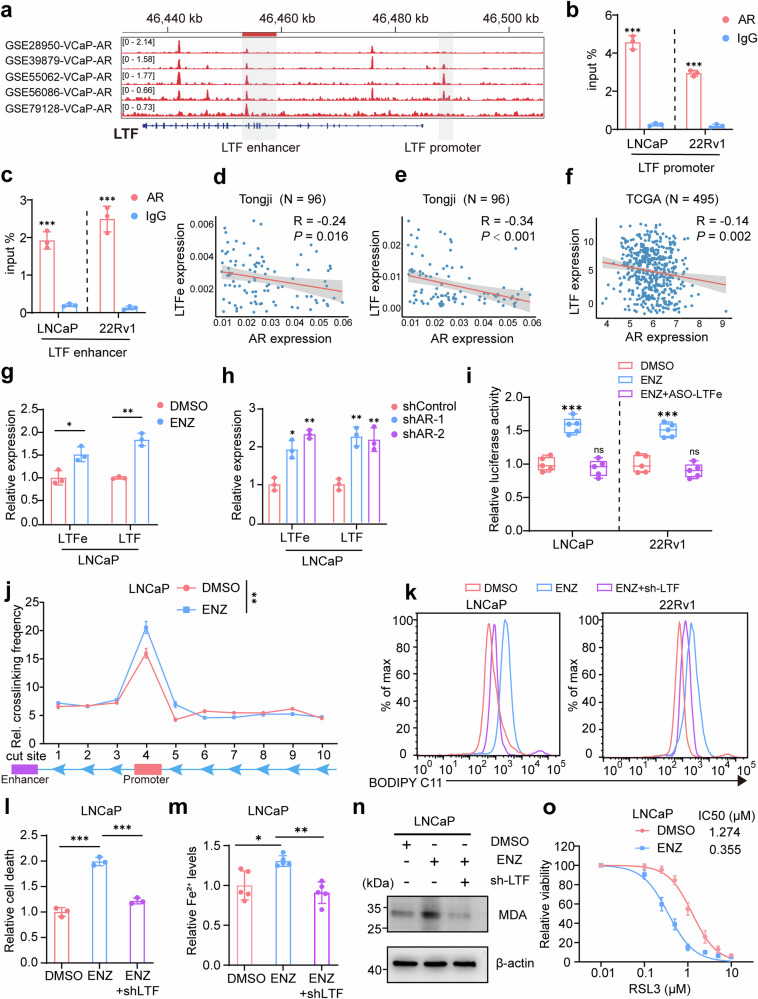
AR signaling inhibits ferroptosis via the LTFe-LTF axis. **a** Visualization of androgen receptor (AR) binding to the *LTF* promoter and enhancer regions, as identified in ChIP-seq datasets (GSE28950, GSE39879, GSE55062, GSE56086, GSE79128). **b**, **c** ChIP-qPCR validation of AR binding to the *LTF* promoter and enhancer regions in LNCaP cells, confirming the interaction observed in ChIP-seq data. **d** Correlation analysis between *AR* and *LTF*e mRNA levels in our prostate cancer samples, presented as Pearson correlation coefficients. **e** Correlation analysis of *AR* and *LTF* mRNA levels in our prostate cancer samples, demonstrating the inverse relationship between *AR* and *LTF* expression. **f** Correlation of AR and *LTF* mRNA levels in the TCGA prostate cancer cohort, expressed as log_2_(TPM + 1), further supporting the negative correlation. **g** Relative *LTF* mRNA levels in LNCaP cells following treatment with enzalutamide (ENZ) (LNCaP: 5 µM ENZ for 48 h) analyzed by qRT-PCR. **h** qRT-PCR analysis of *LTF* mRNA levels in LNCaP cells following AR knockdown, showing an increase in *LTF* expression. **i** Dual-luciferase assays measuring changes in *LTF* promoter activity in LNCaP and 22Rv1 cells treated with DMSO, ENZ, or ENZ + ASO-*LTF*e, indicating the repressive role of AR on *LTF* transcription. **j** 3 C assays showing enhanced interaction frequency between the enhancer and promoter of *LTF* in LNCaP cells treated with ENZ, highlighting the disruption of chromatin looping by AR. **k** Measurement of LipROS levels in LNCaP and 22Rv1 cells treated with DMSO, ENZ, or ENZ+sh*LTF*, reflecting changes in oxidative stress associated with ferroptosis. **l–n** Analysis of cell death rates (**l**), Fe^2+^ levels (**m**), and MDA levels (**n**) in LNCaP cells following treatment with DMSO, ENZ, or ENZ + sh*LTF*, demonstrating the impact of AR signaling on ferroptosis. **o** Cell viability assays in LNCaP cells pre-treated with 5 µM ENZ for 48 h, followed by induction with varying concentrations of RSL3 for 24 h, showing enhanced sensitivity to ferroptosis upon AR inhibition. *P < 0.05, **P < 0.01, ***P < 0.001. Data are presented as mean ± SD, *n* = 3 biologically independent replicates in (**b**), (**c**), (**g**), (**h**), (**j**), (**l**), (**m**) or *n* = 5 biologically independent replicates in (**i**), (**o**). Statistical analysis is performed using one-way ANOVA in (j), and two-sided *t* test in (**b**), (**c**), (**g**), (**h**), (**i**), (**l**), and (**m**)

To test this, we first analyzed the regulatory relationship between AR and LTF by examining androgen response elements (AREs) in the enhancer and promoter regions of the *LTF* gene. Leveraging ChIP-seq datasets (GSE28950, GSE39879, GSE55062, GSE56086, and GSE79128), we identified significant AR binding at these sites (Fig. 7a), suggesting direct AR regulation of the LTFe-LTF axis. This was further confirmed by ChIP-qPCR, where AR binding to these AREs was validated (Fig. 7b, c).

Analysis of multiple prostate cancer cohorts, including our in-house tissue collection (Tongji) and publicly available datasets from TCGA, GEO, and MSKCC, consistently showed a significant negative correlation between AR expression and both *LTF*e and *LTF* expression (Fig. 7d, f, Supplementary Fig. 8a, b). Furthermore, *LTF* expression was significantly lower in samples with AR amplification, suggesting that AR inhibits LTFe-LTF expression, potentially suppressing ferroptosis (Supplementary Fig. 8c).

To explore whether AR inhibition could reverse this effect, we treated prostate cancer cells with the anti-AR drug enzalutamide (ENZ). This led to a significant upregulation of *LTF*e and *LTF* expression, confirmed by qPCR (Fig. 7g, Supplementary Fig. 8d). Inducible AR knockdown similarly elevated *LTF*e and *LTF* expression levels (Fig. 7h, Supplementary Fig. 8e, f). Conversely, treatment with dihydrotestosterone (DHT) suppressed *LTF*e and *LTF* expression, suggesting that androgen signaling via DHT negatively regulates these genes in prostate cancer cells (Supplementary Fig. 8g). Dual luciferase assays demonstrated that ENZ treatment increased *LTF* promoter activity, which was dependent on *LTF*e expression, as knockdown of *LTF*e reversed this effect (Fig. 7i). Additionally, 3C assays revealed enhanced chromatin looping between the *LTF* enhancer and promoter following ENZ treatment, further promoting *LTF* transcription **(**Fig. 7j, Supplementary Fig. 8h).

To directly assess the role of AR in modulating ferroptosis,^32,33^ we measured LipROS levels, a key marker of ferroptosis. ENZ treatment significantly increased LipROS levels, an effect reversed by *LTF* knockdown, indicating that LTF is a crucial mediator of ENZ-induced ferroptosis (Fig. 7k). Further, ENZ treatment increased other markers of ferroptosis, including Fe2+ content, malondialdehyde (MDA) levels, and cell mortality (Fig. 7l–n, Supplementary Fig. 8i–k), all of which were diminished by LTF knockdown. Importantly, ENZ treatment enhanced the sensitivity of prostate cancer cells to RSL3, a selective ferroptosis inducer, indicating that AR inhibition potentiates ferroptosis (Fig. 7o, Supplementary Fig. 8l). Interestingly, RSL3 also resensitized 22Rv1 cells to ENZ treatment, highlighting a bidirectional synergistic effect (Supplementary Fig. 8m).

In summary, our results demonstrate that AR signaling suppresses ferroptosis by downregulating the *LTF*e-*LTF* axis. Targeting AR could enhance ferroptosis in prostate cancer cells, especially for cases resistant to conventional therapies.

### Combination of RSL3 and Enzalutamide as an effective treatment for castration-resistant prostate cancer

Ferroptosis, a form of programmed cell death driven by lipid peroxidation, has emerged as a promising target in cancer therapy. RSL3, a potent ferroptosis inducer, has gained considerable attention in prostate cancer treatment due to its ability to selectively kill cancer cells by targeting the glutathione peroxidase 4 (GPX4) pathway.^34^ Given our previous findings that AR inhibition enhances sensitivity to ferroptosis via the *LTF*e-*LTF* axis, we investigated whether combining RSL3 with the AR antagonist ENZ could provide a more effective therapeutic approach against both hormone-sensitive prostate cancer (HSPC) and CRPC.

**Fig. 8:**
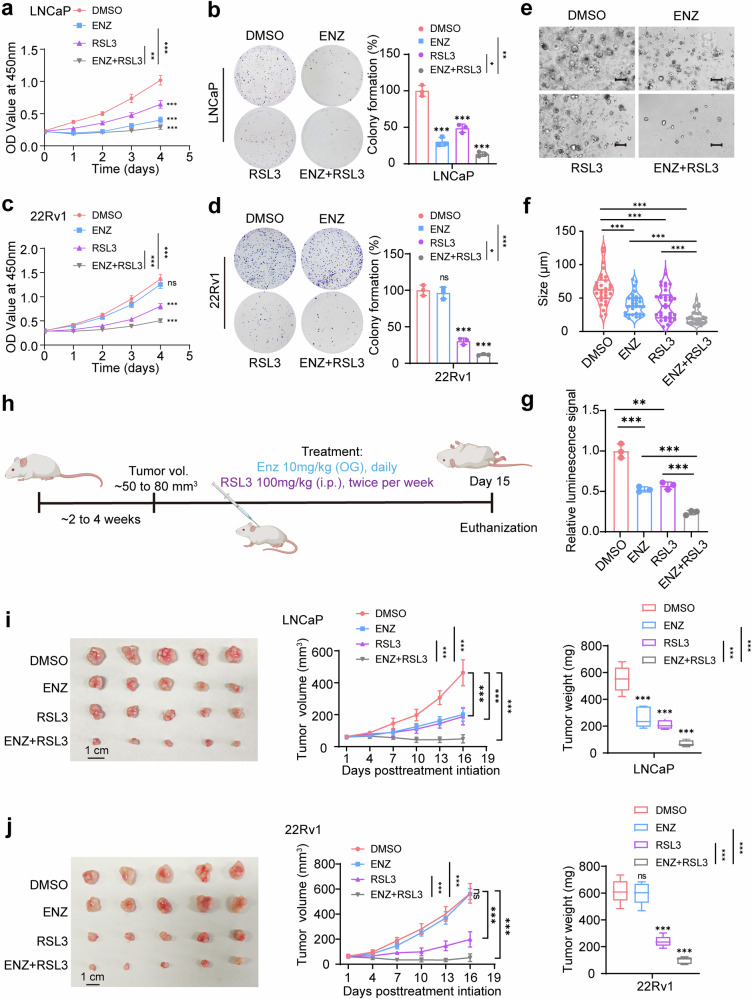
Combining RSL3 and enzalutamide is effective for the treatment of castration-resistant prostate cancer. **a** CCK-8 assays demonstrated that the combination of RSL3 (1 μM) and enzalutamide (ENZ, 5 μM) significantly reduced the growth rate of LNCaP cells compared to either treatment alone. The media were refreshed daily to maintain the effectiveness of the compounds. **b** Colony formation assays in LNCaP cells treated with RSL3 (1 μM), ENZ (5 μM), or a concentration of both. Cells were grown for 14 days, with media containing the respective compounds being refreshed every 3 days. Note that the combination treatment significantly inhibited colony formation compared to single treatments. **c** CCK-8 assays in 22Rv1 cells showed that the combination of RSL3 (1 μM) and ENZ (10 μM) was more effective at reducing cell viability compared to individual treatments. **d** Colony formation assay in 22Rv1 demonstrating that the combination of RSL3 (1 μM) and ENZ (10 μM) significantly suppressed colony formation compared to single treatments. **e** Representative patient-derived organoid images treated with ENZ (5 μM) or RSL3 (1 μM) for 14 days. Scale bar, 100 μm. **f** Quantification of organoid size (*n* = 10 fields from three representative experiments). **g** ATP levels (Luminescence signal) of organoid treated with indicated drugs were shown. **h** Schematic representation of the prostate cancer xenograft model used to evaluate the therapeutic efficacy of RSL3 and ENZ. Mice were treated with RSL3 (100 mg/kg, intraperitoneally, twice per week) and ENZ (10 mg/kg, administered via oral gavage daily) after tumor volumes reached 50–80 mm³. **i**, **j** In vivo results showing the effects of RSL3, ENZ, and their combination on tumor growth in LNCaP and 22Rv1 xenografts. A total of 5 × 10^5^ cells, mixed with 80% Matrigel, were implanted bilaterally into the flanks of nude mice. Tumor volumes were measured every three days, and tumor weights were recorded at the end of the 15-day experiment. The combination treatment led to a significant reduction in both tumor volume and weight compared to single treatments. *P < 0.05, **P < 0.01, ***P < 0.001. Data are presented as mean ± SD. *n* = 3 biologically independent replicates in (**a**)–(**e**), or *n* = 5 biologically independent replicates in (**i**), (**j**). Statistical analysis is performed using two-sided *t* test in (**b**), (**d**), (**f**), (**g**), and tow-way ANOVA in (**a**), (**c**), (**i**), (**j**)

We first analyzed the synergy between RSL3 and ENZ in prostate cancer cell lines using the Loewe model via SynergyFinder 3.0 software. The analysis revealed a significant synergistic interaction between RSL3 and ENZ (Supplementary Fig. 9a, b). To further investigate, we assessed the effects of RSL3 and ENZ, both individually and in combination, on prostate cancer cell lines. LNCaP cells were used as a model for HSPC, while 22Rv1 represented CRPC. As anticipated, RSL3 treatment alone significantly inhibited cell proliferation and colony formation in both cell lines. Remarkably, the combination of RSL3 and ENZ exhibited a synergistic effect, resulting in a significantly greater reduction in cell proliferation and colony formation compared to either agent alone (Fig. 8a–d). This synergistic effect was particularly pronounced in the CRPC 22Rv1 cells, which exhibited resistance to ENZ monotherapy but responded robustly to the combination treatment, suggesting that ENZ sensitizes CRPC cells to ferroptosis (Fig. 8a–d).

To further validate these findings, we performed patient-derived organoid assays. Similar to our observations in cell lines, the combination of RSL3 and ENZ significantly reduced organoid growth, outperforming either monotherapy (Fig. 8e-g). This finding highlights the potential translational relevance of combining ferroptosis inducers with AR-targeting agents to improve therapeutic outcomes in prostate cancer.

To assess the therapeutic potential of the RSL3 and ENZ combination in vivo, we established LNCaP (HSPC) and 22Rv1 (CRPC) xenograft models in immunodeficient mice. Tumors were allowed to grow to an average volume 50–80 mm³ before being treated with RSL3 (100 mg/kg, intraperitoneally, twice per week) and ENZ (10 mg/kg, orally, daily) (Fig. 8h). Consistent with our in vitro findings, the combination therapy resulted in a dramatic reduction in tumor growth compared to monotherapy with either RSL3 or ENZ (Fig. 8i, j). Furthermore, safety and tolerability evaluations revealed no toxicity affecting healthy organs (Supplementary Fig. 9c, d). Importantly, the combination therapy was especially effective in the CRPC xenografts, a model known for its resistance to standard AR-targeted therapies. Tumors treated with the combination of RSL3 and ENZ showed the greatest inhibition in growth, suggesting that this regimen could overcome resistance mechanisms in advanced prostate cancer.

## Discussion

Our study provides novel insights into the functional role of eRNAs in prostate cancer by elucidating the critical contribution of the *LTF*e-*LTF* axis to tumor progression and ferroptosis regulation. While eRNAs have long been recognized as hallmarks of active enhancers, their specific roles in tumorigenesis remain poorly defined.^35^ Using an integrated epigenomic approach, we systematically profiled the eRNA landscape in prostate cancer tissues and identified LTFe as a key regulatory element involved in tumor suppression. Mechanistically, LTFe promotes ferroptosis by facilitating chromatin looping and activating the transcription of its target gene, LTF, a well-established iron-binding protein. Our findings further demonstrate that AR signaling negatively regulates this axis, disrupting enhancer-promoter interactions and impairing ferroptosis sensitivity in prostate cancer cells. Importantly, we show that targeting AR signaling enhances ferroptotic cell death, providing a compelling rationale for combinatorial therapeutic strategies in CRPC. These findings significantly advance our understanding of eRNA-mediated enhancer regulation and suggest a novel therapeutic approach for overcoming resistance to AR-targeted therapies in advanced prostate cancer.

Our findings demonstrate that *LTF*e is a functionally relevant eRNA that regulates prostate cancer progression by modulating enhancer-promoter interactions and promoting ferroptosis. We demonstrate that LTFe recruits HNRNPF, facilitating the formation of chromatin loops between the enhancer and promoter regions of the *LTF* gene. This enhancer-promoter interaction is essential for maintaining LTF transcriptional activity, which in turn promotes ferroptosis by increasing intracellular iron levels and lipid reactive oxygen species (LipROS). Our findings are consistent with previous reports that eRNAs play an active role in stabilizing chromatin contacts and recruiting transcriptional machinery.^13–15^ Notably, we identified AR signaling as a suppressor of this axis, whereby AR binds to both the enhancer and promoter regions of *LTF*, disrupting the chromatin interactions necessary for *LTF*e-driven transcription. This previously unrecognized mechanism highlights the complexity of AR-mediated transcriptional repression in prostate cancer and suggests that targeting AR signaling may restore LTFe function, thereby enhancing ferroptosis sensitivity in CRPC.

Recent studies have highlighted the central role of eRNAs in regulating enhancer-promoter interactions, contributing to tumor development and progression.^36^ For example, Li et al. demonstrated that eRNAs reinforce estrogen-dependent enhancer–promoter looping in breast cancer cells,^37^ while CCAT1, an eRNA associated with colon cancer, was shown to enhance c-MYC expression.^38^ Despite growing interest in eRNAs, their specific roles in prostate cancer have remained largely unexplored. Our study fills this gap by identifying hundreds of differentially expressed eRNAs that are involved in critical cancer-related pathways and are enriched in RBPs). This comprehensive profiling lays the foundation for the development of eRNA-based therapeutic strategies in prostate cancer, marking an important advancement in the understanding of enhancer transcription regulation in this disease.

Ferroptosis is an emerging target in cancer therapy due to its reliance on iron-dependent lipid peroxidation, a pathway that is heavily influenced by iron metabolism.^31,39^ The role of *LTF* in ferroptosis is particularly intriguing given its dual functions in iron regulation and oxidative stress response.^40^ LTF encodes lactotransferrin, an iron-binding protein involved in iron transport and storage, which is critical for maintaining cellular iron balance. Elevated *LTF* expression increases the labile iron pools, creating a cellular environment conductive to ferroptosis through heightened lipid peroxide generation.^40^ This study is the first to implicate an eRNA in ferroptosis regulation, demonstrating that LTFe promotes ferroptosis by upregulating LTF.^31,40^ By enhancing *LTF* expression, *LTF*e increases intracellular iron levels and lipid reactive oxygen species (LipROS), sensitizing prostate cancer cells to ferroptosis. These findings not only expand the understanding of ferroptosis regulation but also suggest that the *LTF*e-*LTF* axis represents a promising therapeutic target for inducing ferroptosis in prostate cancer.

AR signaling plays a central role in prostate cancer development and progression, and AR-targeted therapies such as enzalutamide (ENZ) have become standard treatments.^41^ ENZ inhibits the AR signaling pathway, which regulates key processes including lipid metabolism, oxidative stress, and iron homeostasis, all of which influence ferroptosis regulation.^32,42^ While our findings highlight *LTF* as a central player, the broader effects of ENZ on ferroptosis likely arise from its modulation of AR-dependent pathways. For example, Liang et al. reported that ENZ reduced MBOAT2 expression, thereby enhancing ferroptosis sensitivity through phospholipid profile remodeling.^32^ In addition, ENZ-mediated AR inhibition had been shown to downregulate SLC7A11, a key component of the cystine/glutamate antiporter system, further increasing vulnerability to ferroptosis.^43^ Despite these advancements, resistance to AR inhibition, particularly in CRPC, remains a significant clinical challenge.^44,45^ Our study offers a novel strategy for overcoming this resistance by demonstrating that AR inhibition activates the LTFe-LTF axis, sensitizing prostate cancer cells to ferroptosis. The combination of ENZ and the ferroptosis inducer RSL3 was particularly effective in reducing tumor growth in CRPC models, suggesting that targeting AR signaling can potentiate ferroptosis, thereby offering a promising therapeutic avenue for advanced prostate cancer.

While our findings highlight the therapeutic potential of targeting the *LTF*e-*LTF* axis, several challenges remain. One key limitation of this study is the uncertainty regarding whether *LTF*e selectively regulates *LTF* or influences a broader transcriptional network. Our transcriptomic analysis suggests that LTFe may modulate additional downstream targets, as evidenced by the co-downregulation of several genes in Fig. 3C. However, distinguishing direct from indirect effects remains a complex challenge. Further research involving larger cohorts and advanced sequencing technologies will be required to elucidate the broader regulatory networks of *LTF*e and other eRNAs. Moreover, although targeting eRNA-protein or enhancer-promoter interactions holds therapeutic promise, the clinical application of such strategies is still in its early stages. Future efforts should focus on developing small-molecule modulators that can specifically target eRNAs and their associated chromatin structures, paving the way for eRNA-based therapeutics in cancer. Additionally, this study reveals that LTFe-LTF axis exerts tumor-suppressive effects in primary prostate cancer, while also demonstrating that its expression is repressed by AR. This finding raises the compelling possibility that LTFe expression may be dynamically regulated during ADT, potentially playing distinct roles in the transition to CRPC. Notably, while AR signaling inhibition is a cornerstone of prostate cancer therapy, compensatory mechanisms driving disease progression remain poorly understood. Whether LTFe reactivation under ADT confers tumor-suppressive or pro-tumorigenic functions in CRPC remains an open question. Future research should prioritize longitudinal patient-derived models, single-cell epigenomics, and in vivo CRPC models to determine how LTFe expression dynamics contribute to disease progression and whether LTFe targeting could provide a novel therapeutic vulnerability in castration-resistant settings.

In conclusion, our study uncovers the critical role of the *LTF*e-*LTF* axis in prostate cancer and provides a mechanistic framework for understanding how enhancer RNAs regulate ferroptosis and tumor suppression. By demonstrating that AR inhibition can restore *LTF*e and *LTF* activity, we propose a novel therapeutic strategy that combines AR inhibitors with ferroptosis inducers to effectively treat CRPC. The combination of ENZ and RSL3 shows great promise in overcoming resistance and improving clinical outcomes, warranting further investigation in clinical trials.

## Materials and methods

### Human tissues collection

Prostate cancer tissues and corresponding adjacent normal tissues were obtained from 96 patients at the Department of Urology, Tongji Hospital, Huazhong University of Science and Technology, Wuhan, China. From these, twenty pairs were selected for analysis using ATAC-seq and RNA-seq (Supplementary Table 2, Supplementary Fig. 10a, b). The remaining samples were utilized for validation via quantitative reverse transcription PCR (qRT-PCR) (Supplementary Table 3). All tissue samples were promptly preserved in liquid nitrogen immediately following radical prostatectomy. Informed consent was obtained from all participants, and the study protocol was approved by the Ethics Committee of Tongji Medical College, Huazhong University of Science and Technology (Approval No. 2019CR101).

### ATAC-seq analysis

ATAC-Seq was performed on twenty pairs of prostate cancer and adjacent normal prostate tissues by Seqhealth Technology (Wuhan, China). Approximately 500 mg of each tissue sample was ground into a fine powder and lysed to isolate nuclei by centrifugation at 2000 × *g* for 5 min. Transposition and sequencing libraries were prepared using the TruePrep DNA Library Prep Kit V2 for Illumina (TD501; Vazyme Biotech). These libraries were then enriched, quantified, and sequenced on an Illumina NovaSeq 6000 using the PE150 strategy.

Raw sequence data were initially processed with Trimmomatic (v0.36) to remove adapters and low-quality sequences, followed by duplicate removal using FastUniq (v1.1). Clean reads were aligned to the reference genome using Bowtie2 (v2.2.6) under default settings. Read distribution was analyzed with RSeQC (v2.6), and insert lengths were evaluated using the Collect Insert Size Metrics tool from Picard (v2.8.2). Peak calling and annotation were performed using MACS2 (v2.1.1) and BEDTools (v2.25.0), respectively. Sequencing quality details are provided in Supplementary Table 4.

### Identification of tissue-specific peaks

Building on prior analyses, we further explored chromatin dynamics across individual samples to uncover biologically significant phenomena. MAnorm2 (v1.2.2)^46,47^ was employed to normalize and compare ATAC-seq signals between individual samples or groups, providing a robust framework for quantifying ATAC-seq signals and identifying group-specific patterns. Tissue-specific peaks were determined through a series of analytical steps, including signal normalization, construction of biological conditions (Normal and Tumor), restriction to autosomal regions, modeling a mean-variance relationship, and conducting differential analysis with the diffTest function. Peaks were classified as tissue-specific if they exhibited a |fold change| > 1.5 and a *p*-value < 0.05 in the differential analysis based on M and A values, where M indicates the log2 fold change and A represents the average log2 read count. Functional annotation and pathway enrichment analyses for tumor-specific and normal-specific peaks were conducted using GREAT (http://great.stanford.edu/public/html/).^19^

### RNA-seq analysis

RNA-seq was conducted on prostate cancer and adjacent normal tissues from twenty patients **(**Supplementary Table 2**)**, with experimental procedures and data analysis carried out by Seqhealth Technology (Wuhan, China). For each sample, 2 µg of RNA was used to construct stranded RNA-sequencing libraries using the Ribo-off rRNA Depletion Kit (Illumina, California, USA) and the KCTM Stranded RNA Library Prep Kit for Illumina® (Seqhealth Co., Ltd., Wuhan, China).

The quality of RNA-seq data was evaluated with FastQC (v0.11.9) (www.bioinformatics.babraham.ac.uk/projects/fastqc/). Reads with poor quality were filtered, and adapter sequences were eliminated using Trim Galore (v0.6.7) (https://www.bioinformatics.babraham.ac.uk/projects/trim_galore/). High-quality sequences were then aligned to the human reference genome (hg38) through STAR (v2.7.9a),^48^ and the resulting BAM files were sorted with SAMtools (v1.13).^49^ Gene-level read counts and normalized TPM expression values were generated by StringTie (v2.2.1).^50^ The limma package (v3.46.0) was employed for batch effect correction and the identification of DEGs. Genes with | log2(fold change)| > 1 and FDR < 0.05 were classified as DEGs.

### eRNA identification

ATAC peaks located within ± 2.5 kb of the transcription start site (TSS) were excluded, and the remaining ATAC peaks were categorized as enhancers. Following Zhao et al.^51^, transcripts overlapping with protein-coding genes, antisense transcripts, and other RNA types (rRNA, snRNA, miRNA, etc.) were excluded. Transcripts with an average expression (fragments per kilobase million, FPKM) greater than 1 were classified as putative eRNAs. Consistent with Zhang et al.^26^, regions extending ± 3 kb from the center of annotated enhancers were defined as potential eRNA-transcribing areas. Strand-specific read counts were normalized for each transcript using HOMER, and differential expression analysis was conducted using the EdgeR package, identifying transcripts with over 1.5-fold changes and *P* value < 0.05.

### ChIP-seq Processing

Histone ChIP-seq libraries underwent sequencing, generating 150 bp paired-end reads. The quality of the sequencing output was evaluated using FastQC (v0.11.9), which provided comprehensive quality assessment reports. To process the data, TrimGalore (v0.6.7, RRID: SCR_011847) was utilized for removing adapter sequences and short reads. The refined reads were mapped to the hg38 human reference genome using Bowtie2 (v2.2.5)^52^ under default parameters. Low-quality alignments were filtered with SAMtools (v1.9)^53^ using the options “-q 30 -F 3844”. Duplicate reads were identified and excluded with the Picard toolkit (v2.25.1, RRID: SCR_006525). Quality metrics, including enrichment assessment without peak calling, were further examined using ENCODE’s Phantompeakqualtools,^54^ with fragment length determined by the run_spp.R script. Peak regions were identified through MACS3 (v3.0.0a6)^55^ using the parameters “--nomodel --shift 0 --extsize fragment_length” and a significance cutoff of *p* < 1e-3. The resulting peaks were visualized and interpreted using the Integrated Genome Viewer (IGV, v2.12.3).

### Enhancer region identified from H3K27ac ChIP-seq data

Enhancer regions derived from H3K27ac ChIP-seq data were identified for each sample using the ROSE algorithm.^56^ A maximum linking distance of 12.5 kb was applied for stitching (-s), and regions located within ±2500 base pairs of transcription start sites (-t) were excluded to avoid promoter-associated biases in the analysis.

### RNA binding proteins prediction

RBPmap (https://rbpmap.technion.ac.il/)^57^ was employed to predict binding proteins associated with 1428 validated eRNAs. FASTA sequence files were first generated from eRNA regions using bedtools (v2.26.0),^58^ with the human reference genome set to hg38. These sequence files were then processed in RBPmap, selecting all available human and mouse motifs to identify potential protein binding sites. The motif mapping was conducted using the Weighted-Rank algorithm. A “high” stringency level was applied to enhance result rigor, while other parameters were left at their default values. Predicted results were compiled to quantify the number of each protein on the eRNAs, with significant findings highlighted.

### Prediction of LTFe structure

The secondary structure of LTFe was predicted using RNAfold (http://rna.tbi.univie.ac.at/cgi-bin/RNAWebSuite/RNAfold.cgi),^21^ which provided base-pair probabilities and dot-bracket notation. Detailed results are included in Supplementary Table 1. To further model the 3D structure of LTFe, we employed 3dRNA (http://biophy.hust.edu.cn/new/3dRNA).^22^

Molecular docking analysis was performed using the HDOCK server (http://hdock.phys.hust.edu.cn/). The structure of HNRNPF was retrieved from the UniProt database (https://www.uniprot.org/) and predicted using AlphaFold2. Visualization of the docking results was carried out using PyMOL software (v 3.1).

### Organoid models

Prostate cancer tissue from patients was collected, dissected, and minced into small fragments. The tissue was enzymatically dissociated using Prostate Cancer Organoid Growth Medium (OGM, DAXIANG, China) supplemented with TrypLE (Gibco) and Y-27632. Luminal cells were then isolated via fluorescence-activated cell sorting (FACS, Aria II, BD). The isolated cell pellet was resuspended in prostate cancer organoid culture medium (DAXIANG, #:OC100139) and combined with Matrigel at a 1:2 ratio. To prevent premature polymerization, the cell-Matrigel mixture was maintained on ice. A 50 μL aliquot of the suspension was seeded into each well of a 24-well plate and allowed to solidify at 37 °C for 10 min. Once solidified, 500 μL of organoid culture medium supplemented with anti-apoptotic factors was added to each well. Organoid cultures were maintained with media changes every 3–4 days, and anti-apoptotic factors were omitted from subsequent media replacements. Organoid growth and morphology were regularly monitored. Organoid formation assays were initiated with 2000 cells per well, and organoid development was evaluated on day 14 based on size, morphology, and structural characteristics.

For the organoid viability assay, organoids were cultured in 96-well plates and exposed to drugs as specified. Fresh medium was replaced every other day, and ATP levels were measured using CellTiter-Glo 3D Reagent (Promega).

### Cell culture

The LNCaP, 22Rv1, DU145, C4-2, PC-3, RWPE-1, and 293T cell lines were obtained from the Shanghai Cell Bank Type Culture Collection Committee (Shanghai, China). LNCaP, 22Rv1, C4-2, PC-3, and DU145 cell lines were cultured in RPMI-1640 medium, 293T in DMEM, and RWPE-1 in Keratinocyte-SFM (1×) liquid (Invitrogen, USA). All media were supplemented with 10% FBS (Gibco). The cells were incubated at 37 °C in a humidified atmosphere with 5% CO2.

### RNA fluorescence in situ hybridization (RNA-FISH) assay

The biotin-labeled antisense *LTF*e probe was synthesized by RiboBio (Guangzhou, China). FISH assays were performed on prostate cancer cells seeded on coverslips and fixed with 4% paraformaldehyde, following the protocol provided by RiboTM (RiboBio, China). Fluorescence microscopy was used to capture the final images.

### Chromatin isolation by RNA purification (ChIRP) assay

ChIRP assays were conducted using the BersinBio^TM^ chromatin isolation by RNA purification kit (BersinBio, China). Prostate cancer cells (8.0 × 107) were cross-linked and prepared for the assay. Antisense oligonucleotides targeting *LTF*e were designed by LGC Biosearch Technologies, with sequences detailed in Supplementary Table 5.

### Chromosome conformation capture assays

Quantitative analysis of chromosome conformation capture assays (3C-qPCR) was performed in prostate cancer cells. Briefly, 1×10^7^ prostate cancer cells were fixed with 2% formaldehyde for 10 min, and the reaction was quenched with glycine. Cells were lysed in 0.5 mL lysis buffer (10 mM Tris-HCl, pH 7.5; 10 mM NaCl; 0.2% NP-40; 1 × complete protease inhibitor) and digested overnight with the Scal enzyme (New England Biolabs) at 37 °C, followed by ligation with T4 ligase (Thermo Fisher) at 16 °C for over 4 h. Cross-linked DNA fragments were extracted using phenol-chloroform. Amplification efficiency was standardized using a bacterial artificial chromosome (BAC) clone (provided by The Genome Resource Laboratory, Huazhong Agricultural University) encompassing the genome segment of the target regions. Physical interactions between anchor and test primers were quantified by quantitative PCR (qPCR), and values were normalized against *GAPDH*. All 3C-qPCR primers are detailed in Supplementary Table 5.

### RNA extraction and quantitative real-time PCR (RT-qPCR)

Total RNA from cells or tissues was extracted using TRIzol reagent (Invitrogen, USA). Complementary DNA (cDNA) was synthesized via reverse transcription with the Prime-Script™ RT Reagent Kit (Vazyme, China). Quantitative real-time PCR (RT-qPCR) was performed using SYBR Green Mix (Vazyme, China). Gene expression levels were normalized to *GAPDH*. The sequences of all primers used in qPCR are provided in Supplementary Table 5.

### Lentivirus production and stably transfected cell line construction

To produce lentivirus, 293T cells were co-transfected with recombinant lentiviral and packaging plasmids using Lipofectamine 3000 (Invitrogen). The PLKO.1 empty vector was used as a negative control. Following a 48-h incubation, prostate target cells were exposed to the lentivirus-enriched medium. Puromycin (2 mg/mL) facilitated antibiotic selection. Stable transfectant pools were generated and confirmed through RT-qPCR and western blot analysis. Cells that survived the selection process were used in subsequent experiments. Primer details are provided in Supplementary Table 5.

### Western Blot

Protein extraction was performed using RIPA lysis buffer (Beyotime, China) supplemented with 1× Protease Inhibitor Cocktails (Servicebio, China). Subsequently, proteins were separated using either 10% or 8% gradient SDS-PAGE gels and transferred to polyvinylidene fluoride (PVDF) membranes. The membranes were blocked with 5% BSA for 1 h, incubated with primary antibodies overnight at 4 °C, and then with horseradish peroxidase-conjugated secondary antibodies at room temperature for 1 h. Visualization was achieved using an enhanced chemoluminescence detection system. Details of the antibodies are provided in Supplementary Table 6.

### Subcellular fractionation assays

Cell nuclear and cytoplasmic fractions were isolated from cell samples using NE-PER Nuclear and Cytoplasmic Extraction Reagents (Thermo Fisher, USA) following the manufacturer’s protocol. RNA from each fraction was extracted with TRIzol.

### Chromatin immunoprecipitation qPCR (ChIP-qPCR)

ChIP-qPCR assays were conducted using the ChIP assay kit protocol (Cell Signal Technology, USA). Chromatin from fixed prostate cancer cells was isolated and fragmented via sonication in an ice bath. Antibodies targeting H3K27ac, H3K4me1, H3K4me3, HNRNPF, AR, and IgG were used for overnight immunoprecipitation at 4 °C with cross-linked protein/DNA complexes, employing protein A/G magnetic beads. The purified DNA was subsequently quantified using real-time PCR with SYBR Green PCR Master Mix (Vazyme, China). Primers specific to the active enhancer regions are listed in Supplementary Table 5, while the antibodies used are detailed in Supplementary Table 6.

### RNA immunoprecipitation assay

In the RNA immunoprecipitation (RIP) assay, we used anti-HNRNPF and anti-IgG antibodies in conjunction with the Magna RIP Kit (Cat# 17–701, Millipore). Prostate cancer cells cultured in a 15 cm dish were washed twice with 10 mL of pre-chilled PBS. Cells were scraped into 10 mL of PBS, collected by centrifugation at 1500 rpm for 5 min at 4 °C, and then incubated overnight at 4 °C with 10 µg of anti-HNRNPF antibody (Proteintech) bound to magnetic beads, with rotation. Following incubation, the beads were washed five times with RNA immunoprecipitation wash buffer. RNA was then purified using a mixture of phenol, chloroform, and isoamyl alcohol (125:24:1 ratio) along with alcohol and precipitate enhancer. Coprecipitated RNAs were quantified via quantitative RT-PCR, with total RNA and isotype IgG serving as input and negative controls, respectively. Primers used in RIP-qPCR are listed in Supplementary Table 5.

#### RNA half-life assay

Prostate cells were treated with 5 μg/ml Actinomycin (GLPBIO) to inhibit transcription. RNA samples were collected at multiple time points, and the degradation of RNA was assessed by RT-qPCR. The relative expression levels of eRNAs were calculated as the ratios of RNA levels at each time point to the levels at the initial time point and subsequently plotted.

### Luciferase reporter assay

The dual-luciferase assays were conducted using Promega’s Dual-Luciferase Reporter Kit (USA) following the manufacturer’s instructions. We synthesized the human *LTF* promoter and an active enhancer from Generay Biotech (Shanghai, China). These sequences were then subcloned into the PGL3-Basic vector (Promega) to create luciferase reporter plasmids. These plasmids were co-transfected into prostate cancer cells along with the pRL-SV40 Renilla luciferase plasmid (Promega, USA). Luciferase activity for each sample was measured by normalizing the luminescence of firefly luciferase to that of Renilla luciferase.

### CCK-8 and colony formation assays

For the CCK-8 assays, cells were resuspended and seeded at densities of 2000 cells per well for both 22Rv1 and LNCaP in 96-well cell culture plates. Cell viability and proliferation were assessed using the CCK-8 reagent (Dojindo, Japan). In the colony formation assays, we seeded approximately 3000 LNCaP cells and 1000 22Rv1 cells into six-well plates. Following a 14-day incubation period, colonies were stained with crystal violet, and the number of cell colonies was quantified and analyzed.

### Synergy score analysis

To evaluate the synergistic antitumor effects of enzalutamide (ENZ) and RSL3, 22Rv1 and LNCaP cells were treated with specified concentrations of ENZ, either alone or in combination with RSL3. Drug interaction synergy scores were calculated using the Loewe additivity model with SynergyFinder 3.0 software (www.synergyfinder.org).^59^

### BrdU assay

Cell proliferation was assessed using the BrdU Cell Proliferation ELISA Kit (Abcam, Cat#: ab126556). Prostate cancer cells were seeded at a density of 2 × 10^5^ cells/mL in 100 μL of culture media per well. BrdU label (20 μL) was added to designated wells, and cells were incubated for 24 h. Following incubation, 200 μL of Fixing Solution was added to each well and incubated at room temperature for 30 min. After washing, cells were incubated with 100 μL of anti-BrdU antibody per well for 1 h at room temperature. Subsequently, 100 μL of Peroxidase Goat anti-mouse IgG was added and incubated for 30 min at room temperature. Absorbance at 450 nm was measured to evaluate cell proliferation.

### Cell cycle analyses

Cell cycle distribution (G0/G1, S, and G2/M phases) was analyzed using a cell cycle kit (Beyotime). Cells were harvested by trypsinization, fixed in 1 mL of 70% ethanol, and incubated at 4 °C. After fixation, cells were centrifuged at 1000 × *g* for 5 min, resuspended in 500 µL of staining buffer, and treated with 10 µL of RNase A. The mixture was incubated at room temperature, followed by the addition of 25 µL of propidium iodide. After incubation at 37 °C for 30 min, cell cycle distribution was analyzed by flow cytometry.

### Gene set variation analysis (GSVA)

Single-sample gene set variance analysis was performed using the R package ‘GSVA’ (v.1.30.0). This non-parametric approach assesses gene set enrichment to provide an enrichment score for each gene set within a sample. Enrichment scores were derived from log-transformed protein-coding gene-level TPM data for each gene set.

### Lipid reactive oxygen species (LipROS) assay

Tumor cells were seeded at a density of 3 × 10^4^ per well in 24-well plates. Cells were trypsinized, centrifuged, and resuspended in 300 μl of phosphate-buffered saline (PBS) containing 5 μM BODIPY 581/591 C11. They were then incubated at 37 °C for 20 min in a tissue culture incubator, washed, resuspended in 200 μl PBS, and immediately analyzed using a flow cytometer.

### Cell death assay

Tumor cells (3 × 10^4^ cells/well) were plated in 24-well plates. The cells were collected and resuspended in 200 μl PBS with 1 μg/ml Propidium Iodide (PI). This mixture was incubated for 10 min and then assessed immediately on a flow cytometer to determine the percentage of PI-positive (dead) cells.

### Iron assay kit

Iron levels were quantified using the Iron Assay Kit (Abcam, Cat#: ab83366). Briefly, 2 × 10^6^ prostate cancer cells were homogenized in four volumes of Iron Assay Buffer. The homogenate was centrifuged at 16,000 × *g* for 10 min at 4 °C to remove insoluble debris. The resulting supernatant was incubated with the Iron Probe and Assay Buffer for 60 min at 25 °C. Absorbance at 593 nm was then measured to determine the iron concentration.

### Xenograft experiments

Male BALB/c nude mice were sourced from Vital River Laboratory Animal Technology (Beijing, China). For subcutaneous injection experiments, 5.0 × 10^6^ LNCaP cells and 3.0 × 10^6^ 22Rv1 cells were suspended in 100 μL of PBS and injected subcutaneously into the right flank of 6-week-old mice (*n* = 5 per group). ASO was administered directly into the tumor mass every 3 days 8 times when tumor reached approximately 50-100 mm³. In the combination therapy experiments, mice were randomized into various treatment groups: vehicle control, RSL3 (100 mg/kg), enzalutamide (10 mg/kg, dissolved in 5% DMSO, 30% PEG 300, and 65% water, administered via oral gavage daily), and a combination of RSL3 with enzalutamide for 15 days. Tumor volumes were calculated using the formula: Volume = 0.5 × length × width^2^. All animal experiments were approved by the Ethics Committee of Tongji Hospital, Tongji Medical College, Huazhong University of Science and Technology (Approval No. TJH-201901019).

### Statistical analysis

Differential statistics were analyzed using GraphPad Prism 9 software. Data are presented as mean ± standard deviation. Statistical significance between groups was determined using a two-sided Student’s *t*-test. Gene co-expression was assessed using Pearson correlation analysis. Kaplan–Meier survival analysis was performed to evaluate the association between gene expression and prostate cancer patient survival, with *P* values determined by the log-rank test. *P* values < 0.05 were considered statistically significant. In figure legends, *, *P* < 0.05; **, *P* < 0.01; ***, *P* < 0.001, and nonsignificant differences are indicated with ns.

## Supplementary information

Supplementary_Materials

Data S1

## Data Availability

Data supporting this study are available from the corresponding author upon reasonable request. All raw and processed data have been deposited in the Gene Expression Omnibus (GEO) under accession number GSE221109. The RNA-seq and microarray data, including datasets such as Chinese PCa (Cell Res 2012), CPGEA (Nature 2020), TCGA, Fred Hutchinson CRC (Nat Med 2016), Taylor (GSE21034), Yu (GSE6919), Grasso (GSE35988), Chandran (GSE6752), Nanni (GSE3868), Tamura (GSE6811), Varambally (GSE3325), Setlur (GSE8402), Wallace (GSE6956), Bittner (GSE2109), Stockholm, and Camcap (GSE70770), were obtained from public databases, including the cBioPortal for Cancer Genomics and the GEO database.
