# Supplementary material for: Enhancer transcription profiling reveals an enhancer RNA-driven ferroptosis and new therapeutic opportunities in prostate cancer
Source: Signal Transduct Target Ther. 2025 Mar 14;10:87. doi: 10.1038/s41392-025-02170-6 (PMC11906896; doi:10.1038/s41392-025-02170-6)
Supplement: Supplementary file 1 — Supplementary_Materials [file 41392_2025_2170_MOESM1_ESM.docx]

Supplementary Materials for

**Enhancer transcription profiling reveals an enhancer RNA-driven ferroptosis and new therapeutic opportunities in prostate cancer**

Sheng Ma^1＃^, Zixian Wang^2＃^, Ze-Zhong Xiong^1^^＃^, Yue Ge^1＃^, Meng-Yao Xu^1^, Junbiao Zhang^1^, Yuzheng Peng^1^, Qin Zhang^3^, Jiaxue Sun^2^, Zirui Xi^1^, Hao Peng^1^, Wen-Jie Xu^2^, Yanan Wang^1^, Le Li^1^, Chunyu Zhang^1^, Zheng Chao^1^, Baojun Wang^4^, Xu Gao^5^,Xu Zhang^4*^, Gong-Hong Wei^2,6*^, Zhihua Wang^1, 7*^

Correspondence to: zhwang_hust@hotmail.com

**This PDF file includes:**

Figures. S1 to S10

Tables S1 to S6

Captions for Data Sl

**Other Supplementary Materials for this manuscript include the following:**

Data S1 (separate file)


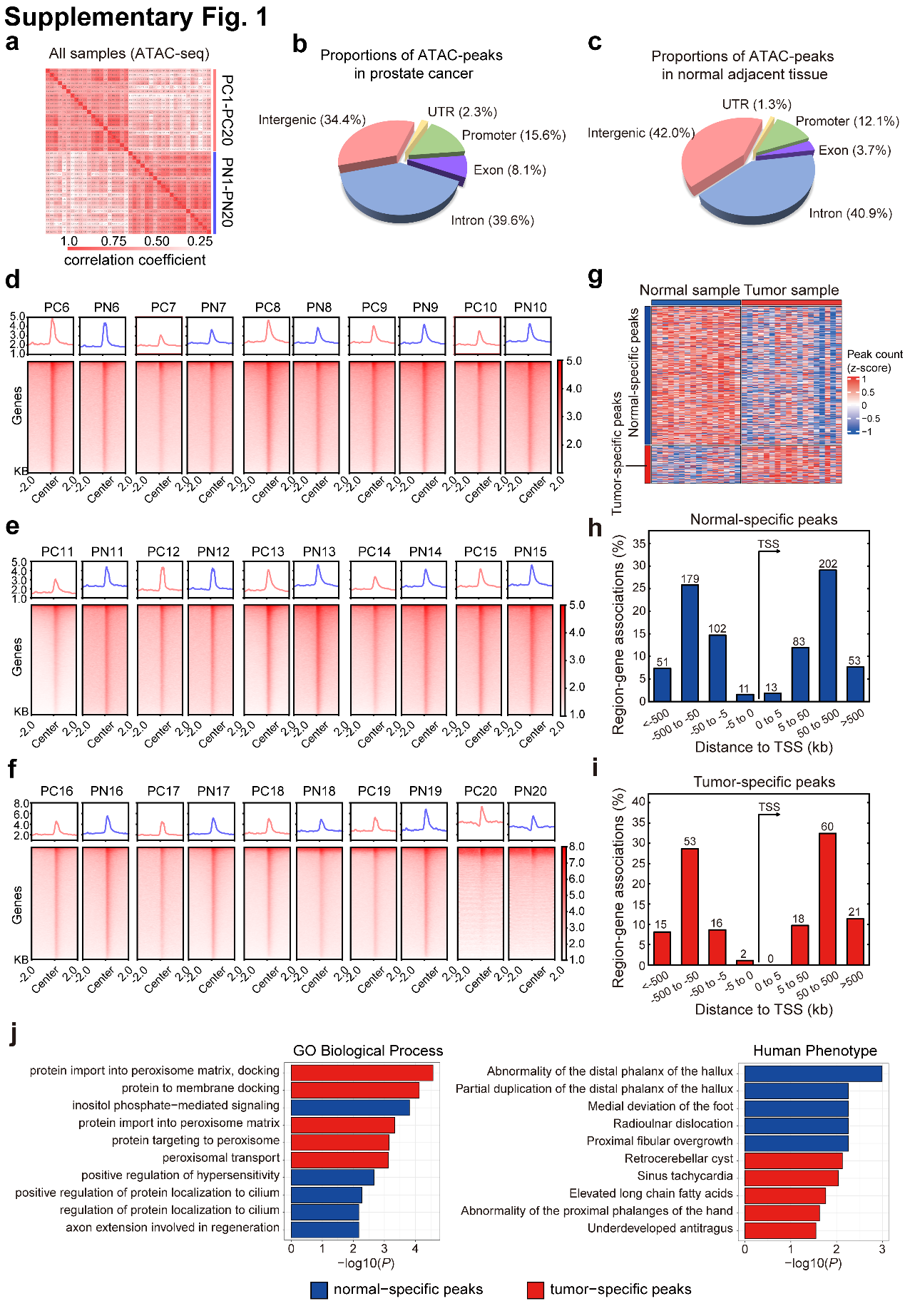


**Supplementary Fig. 1. Characterization of enhancer transcription profile in prostate cancer.**

**(a)** Correlation of chromatin accessibility of 20 pairs of prostate cancer and matched normal prostate samples by ATAC-seq analysis. Values were calculated by Spearman correlation analysis. **(b, c)** Proportions of the ATAC-seq peak regions identified in prostate cancer and normal prostate tissues, which represent the various genome annotations. **(d-f)** Heat map indicating ATAC-seq signal intensity within ± 2 Kb around the center of ATAC peaks in another 15 pairs of prostate cancer and normal prostate tissues. ATAC-seq signals were not displayed outside ± 2 Kb from the nearest ATAC peaks. **(g)** Peak abundance of tissue-specific peaks in normal and tumor samples. Four normal samples and two tumor samples were excluded due to lack of distinct clustering. **(h-i)** Enrichment of normal-specific peaks and tumor-specific peaks near transcription start sites (TSS)**.** **(j)** Pathway enrichment analysis based on normal-specific peaks and tumor-specific peaks, showing the top 5 pathways from Gene Ontology (GO) Biological Process and Human Phenotype Ontology.


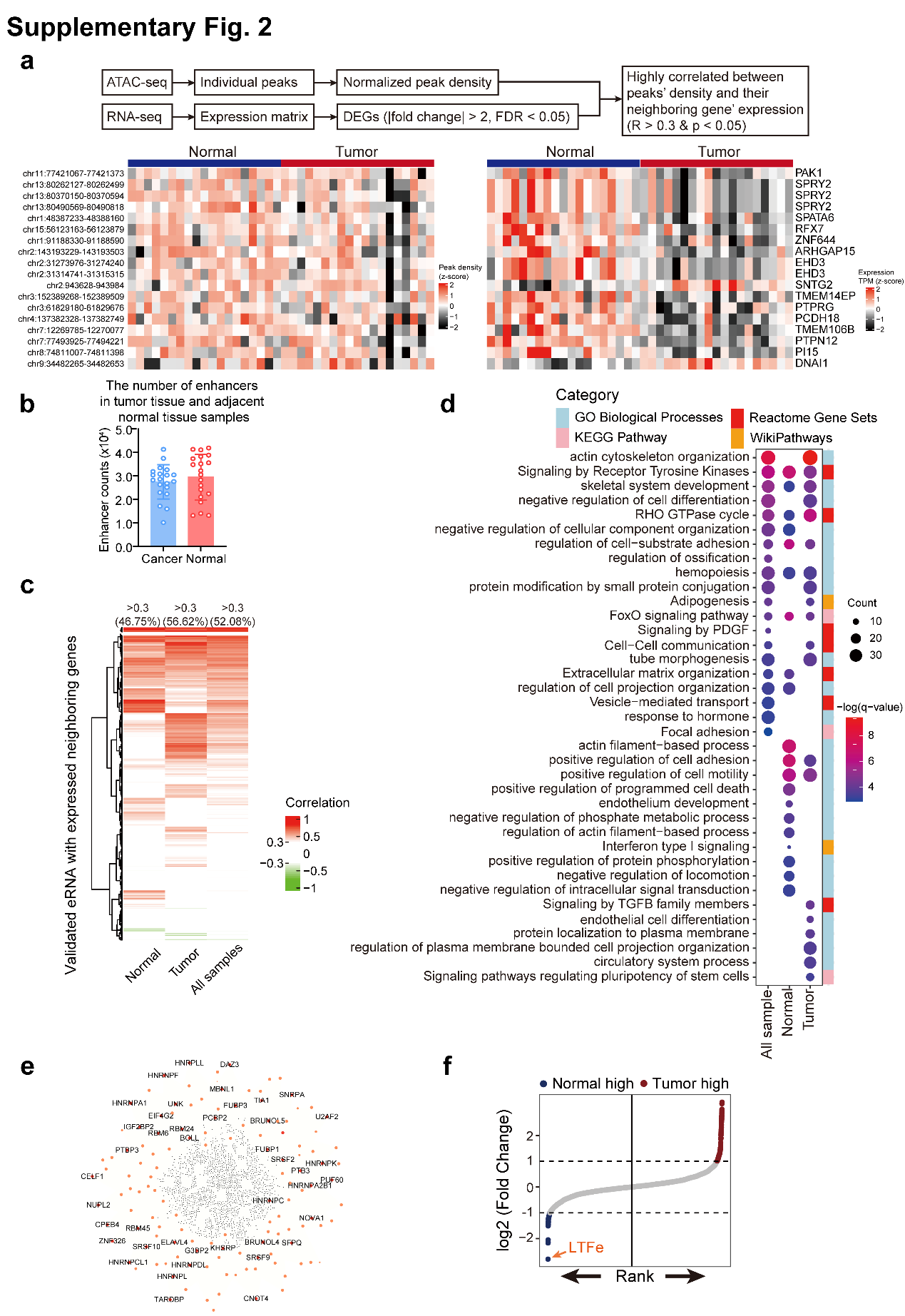


**Supplementary Fig. 2. Analysis of ATAC peaks and neighboring genes.**

**(a)** Spearman correlation analysis showing significant correlations between the normalized signals of identified peaks and the expression levels of their corresponding neighboring genes. Peaks with strong correlations (R > 0.3, *p* < 0.05) were highlighted. **(b)** Quantification of identified enhancers in tumor tissue compared to adjacent normal prostate tissue samples. **(c)** Heatmap displaying the results of Spearman correlation analyses for each eRNA, illustrating its individual correlation with the expression of neighboring genes. **(d)** Pathway enrichment analysis of neighboring genes that exhibited a strong positive correlation with eRNA expression. **(e)** Validated eRNA-RBP prediction network. The black dots at the center of the figure represent the 1,428 validated eRNAs, while RBPs are marked in orange and red. RBPs highlighted in red are predicted to potentially bind to all eRNAs. **(f)** Rank map of differentially expressed eRNAs, highlighting LTFe as the most significantly altered eRNA.

**
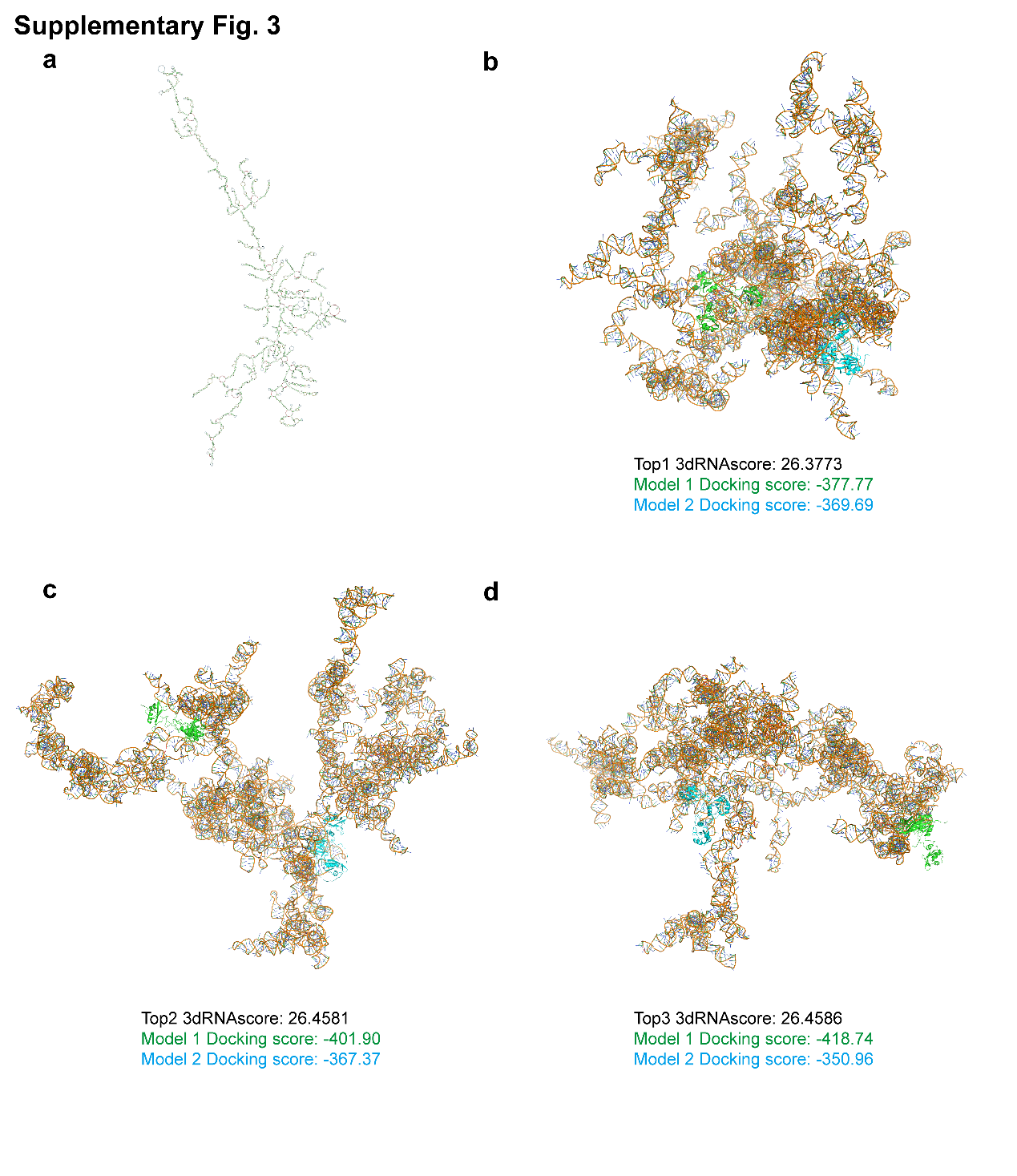
 Supplementary Fig. 3 Predicted structural of LTFe.**

**(a)** Predicted secondary structures of LTFe. **(b-d)** Predicted tertiary structures of LTFe, generated using 3dRNA. Panels (b), (c), and (d) show the top three models ranked by their prediction scores of 26.3773, 26.4581, and 26.4586, respectively. Molecular docking analysis of the RNA-binding protein HNRNPF with the predicted 3D structure is presented in the corresponding panels. The two highest predicted binding sites are highlighted, with Model 1 shown in green and Model 2 in blue. Docking scores were calculated using the HDOCK server.


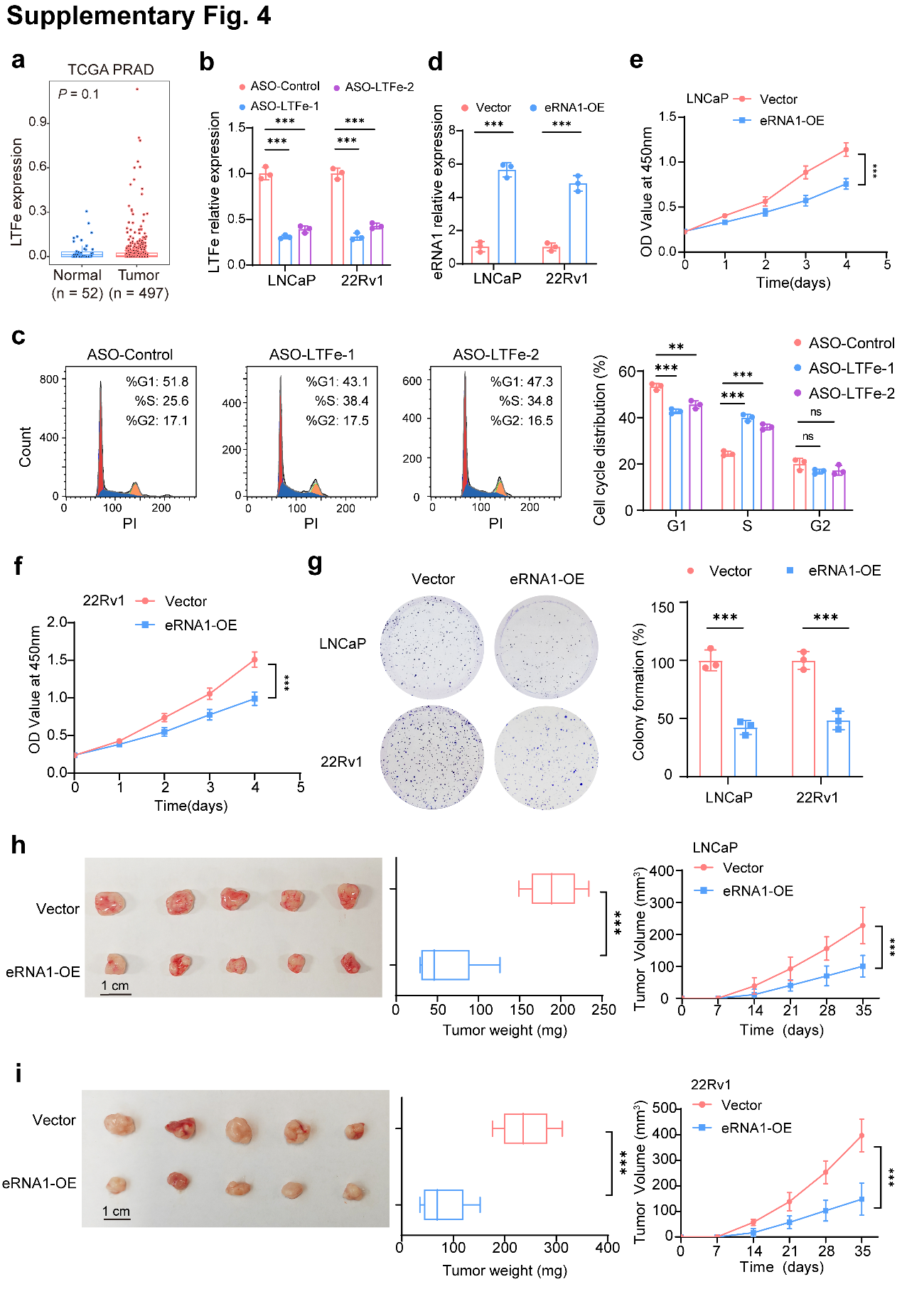


**Supplementary Fig. 4. LTFe influences prostate cancer proliferation both in vitro and in vivo.**

**(a)** Expression difference of LTFe between cancerous and adjacent normal tissues in TCGA dataset. **(b)** Real-time qPCR analysis of LTFe expression in LNCaP and 22Rv1 cells following ASO-LTFe treatment, confirming effective knockdown. **(c)** Flow cytometry analysis of the cell cycle profile in 22Rv1 cells after LTFe knockdown. **(d)** Real-time qPCR analysis showing the relative expression of LTFe in LNCaP and 22Rv1 cells post LTFe-OE transfection. **(e, f)** CCK-8 assays showing that LTFe overexpression markedly reduced the proliferation of prostate cancer cells in vitro. **(g)** LTFe overexpression significantly decreasing the ability of prostate cancer cells to form colonies in vitro. **(h, i)** Tumor volume and weight measurements in xenografts derived from LNCaP and 22Rv1 cells treated with LTFe-OE.

**P* < 0.05, ***P* < 0.01, ****P* < 0.001. Data are presented as the means ± SD, n = 3 biologically independent experiments in (b)-(g) or n = 5 in (h), (i). Statistical analysis is performed using two-sided Student's t-test in (a), (b), (c), (d), (g) and tow-way ANOVA in (e), (f), (h), (i).


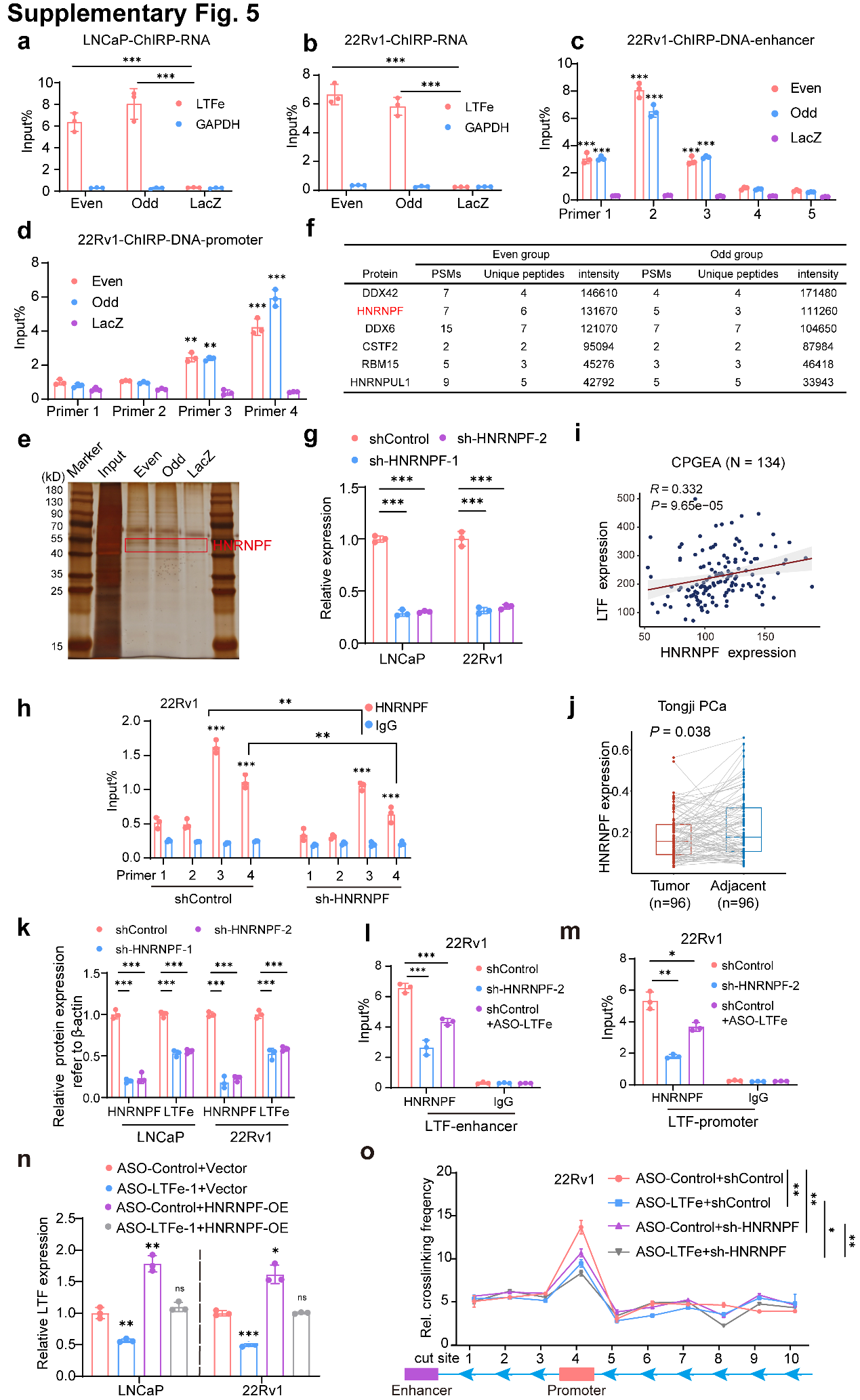


**Supplementary Fig. 5. Identification of LTFe binding protein HNRNPF enhancing LTF transcription through chromatin loop formation.**

**(a, b)** ChIRP assay performed using two different sets of antisense probes (“even group” and “odd group”) that detected *LTF*e or control probes (anti-Lacz) in prostate cancer cell lines. The enrichment of *LTF*e and *GAPDH* mRNA in the ChIRP and input samples was measured by RT-qPCR. **(c, d)** Enrichment of genomic DNA in the ChIRP and input samples quantified using RT-qPCR in 22Rv1, employing six different qPCR primers in *LTF* enhancer and four qPCR primers in *LTF* promoter. **(e)** Silver staining identified the binding of protein complexes to *LTF* in 22Rv1. **(f)** Mass spectrometry identified potential candidate proteins binding to *LTF*e. **(g)** RT-qPCR confirming the HNRNPF expression in LNCaP and 22Rv1 cells transfected with shControl and sh-HNRNPF. **(h)** RIP-qPCR experiments showing significant HNRNPF binding within the *LTF*e region 3, which decreased following HNRNPF knockdown in 22Rv1. **(i)** Correlation analysis demonstrating a positive correlation between HNRNPF and *LTF* expression levels in CPGEA prostate cancer cohort. **(j)** Analysis of prostate cancer tissue samples from our in-house prostate cancer cohort revealed that HNRNPF expression was significantly lower in tumor tissues compared to normal tissues. **(k)** Quantification of Western blot analysis showing LTF protein levels in LNCaP and 22Rv1 cells transfected with shControl or sh-HNRNPF. **(l, m)** ChIP-qPCR assays indicating that HNRNPF binds to the enhancer primer 2 and promoter primer 4 regions of *LTF* in 22Rv1, with binding intensity varying based on the expression levels of HNRNPF and *LTF*e. **(n)** RT-qPCR assays measuring *LTF* expression levels in LNCaP and 22Rv1 cells, comparing cells transfected with shControl, ASO-Control, ASO-*LTF*e, co-transfected with either an empty vector or HNRNPF-OE. **(o)** 3C profile enrichment quantified in 22Rv1 cells transfected with ASO-Control, ASO-*LTF*e, sh-HNRNPF, or combined ASO-*LTF*e and sh-HNRNPF plasmids.

**P* < 0.05, ***P* < 0.01, ****P* < 0.001. Data are presented as the means ± SD, n = 3 biologically independent experiments in (a), (b), (c), (d), (g), (i), (h), (k), (l), (m), (n), (o). Statistical analysis is performed using two-sided Student's t-test in (a), (b), (c), (d), (g), (h), (k), (l), (m), (n) paired sample t-test for (j), and one-way ANOVA for (o).


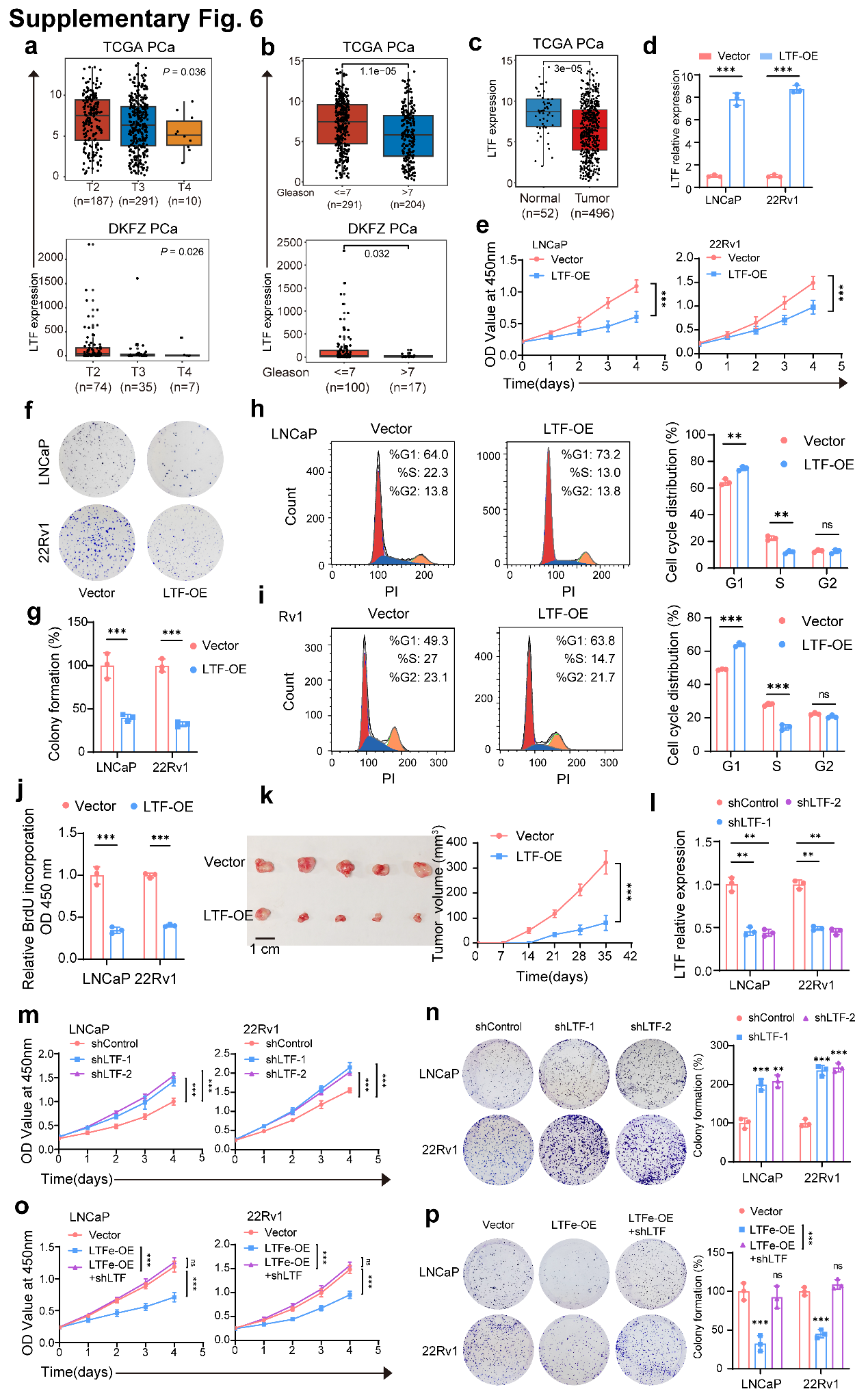


**Supplementary Fig. 6. LTFe modulates cancer proliferation through target gene LTF.**

**(a, b)** In the TCGA and DKFZ cohorts, low *LTF* expression was significantly associated with aggressive disease characteristics, including higher Gleason scores and advanced T stages. **(c)** *LTF* expression is significantly lower in tumor tissues compared to normal prostate tissues, as observed in TCGA cohort. **(d)** qRT-PCR results demonstrating changes in *LTF* expression in LNCaP and 22Rv1 cells following transfection with either a vector or *LTF* overexpression plasmid (*LTF*-OE). **(e)** CCK-8 assays assessing cell viability in LNCaP and 22Rv1 cells stably transfected with either a vector or *LTF*-OE, showing that LTF overexpression reduces cell proliferation. **(f, g)** Colony formation assays showing colony growth in LNCaP and 22Rv1 cells stably transfected with either a vector or *LTF*-OE. **(h, i)** Flow cytometry analysis of the cell cycle profile in LNCaP and 22Rv1 cells after LTF overexpression. **(j)** BrdU assay assessing prostate cancer cell proliferation following LTF overexpression. **(k)** *In vivo* experiments showing that cells overexpressing LTF exhibit significantly slower tumor growth rates compared to the control group. **(l)** RT-qPCR results showing *LTF* expression changes in LNCaP and 22Rv1 cells transfected with either shControl or sh*LTF*. **(m, n)** Stable knockdown of *LTF* significantly stimulated cell proliferation and enhanced colony formation capabilities. **(o, p)** Knockdown of *LTF* counteracting the reduction in cell proliferation triggered by *LTF*e overexpression.

**P* < 0.05, ***P* < 0.01, ****P* < 0.001. Data are presented as the means ± SD, n = 3 biologically independent experiments. Statistical analysis is performed using two-sided Student's t-test in (d), (f), (g), (h), (i), (j), (l), (n), (p) Kruskal-Wallis test in(a), (b), paired sample t-test for (c), and two-way ANOVA for (e), (k), (m), (o).


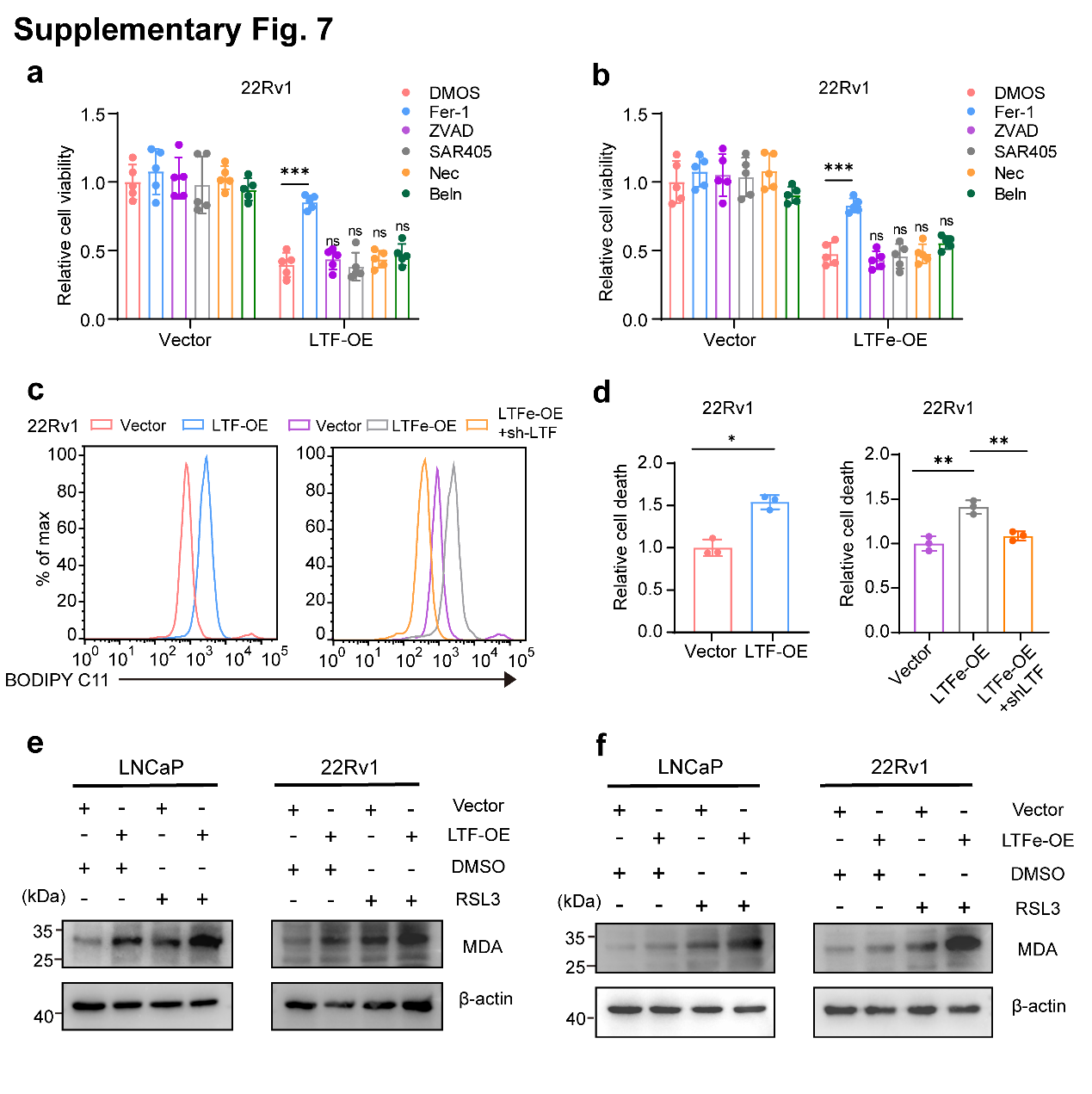


**Supplementary Fig. 7. LTFe-LTF promoted ferroptosis in prostate cancer.**

**(a, b)** Cell viability of 22Rv1 cells transfected with vector, *LTF*-OE or *LTF*e-OE, with and without pretreatment using the ferroptosis inhibitor (Fer-1, 10 μM), the apoptosis inhibitor (ZVAD, 25 μM), the autophagy inhibitor (SAR405, 1 μM), the necroptosis inhibitor (Nec, 25 μM), and the pyroptosis inhibitor (Beln, 25 μM). **(c)** LipROS levels in 22Rv1 cells transfected with vector, *LTF*-OE, *LTF*e-OE, or *LTF*e-OE+sh-*LTF*, assessed via C11-BODIPY fluorescence. **(d)** Cell death rates in 22Rv1 cells transfected with vector, *LTF*-OE, *LTF*e-OE, or *LTF*e-OE+sh-*LTF*, determined by flow cytometry. **(e, f)** Immunoblot analysis of malondialdehyde (MDA) in LNCaP and 22Rv1 cells transfected with vector, *LTF*-OE, or *LTF*e-OE, with and without pretreatment using DMSO or RSL3

The data are presented as the means ± SD, n = 3 biologically independent experiments in (d) or n = 5 in (a) (b). Statistical analysis was performed using two-sided Student's t-test.


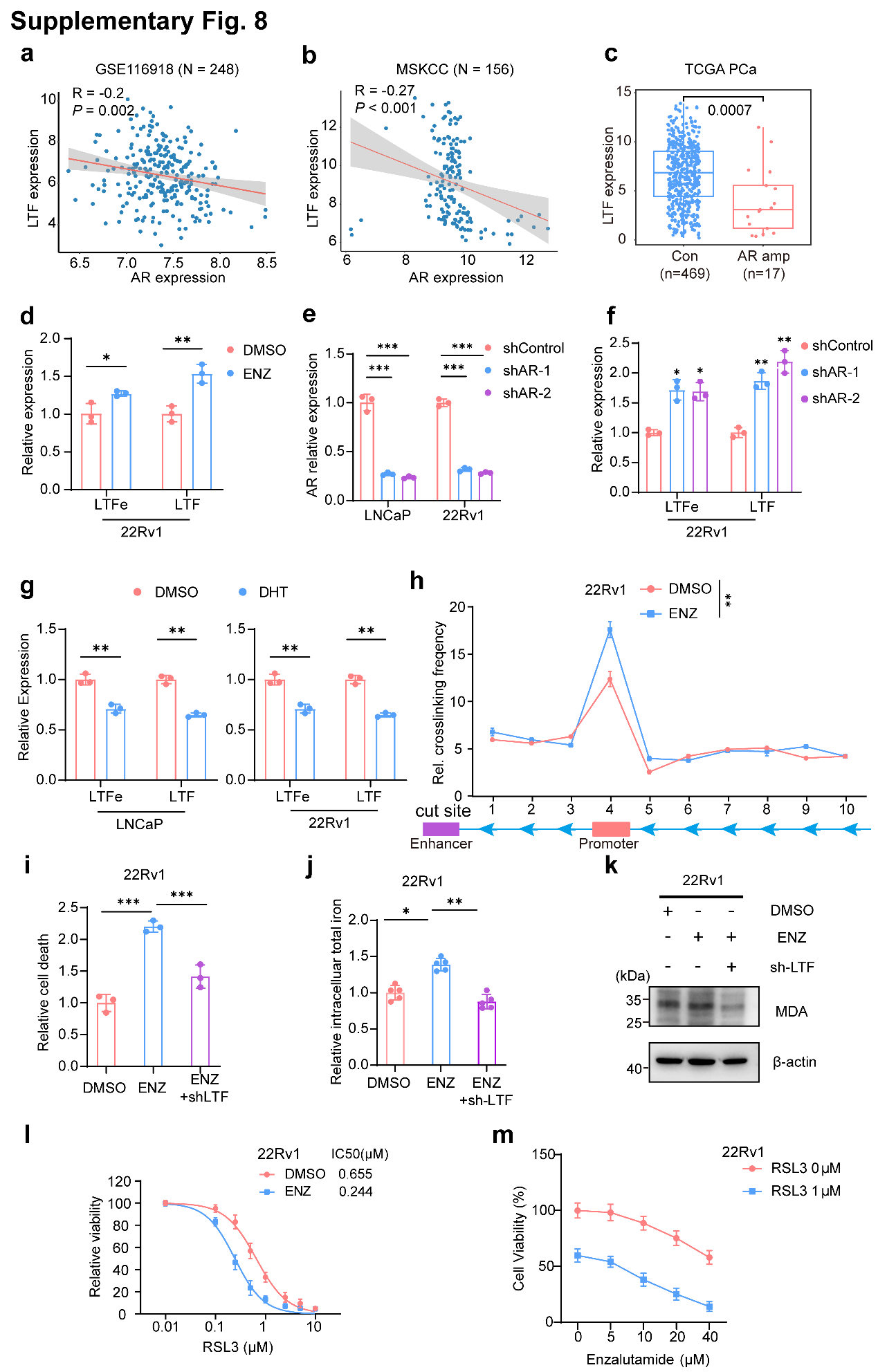


**Supplementary Fig. 8. AR signaling modulated ferroptosis via the LTFe-LTF axis.**

**(a, b)** Correlation between AR and *LTF* mRNA levels in GEO and MSKCC cohorts, expressed as log_2_(RPKM + 1), Pearson correlation coefficient. **(c)** The expression of *LTF* in the AR-amplified group was significantly lower compared to the control group. **(d)** Relative *LTF* mRNA levels in 22Rv1 cells analyzed by qRT-PCR (10 µM ENZ for 72 hours). **(e)** qRT-PCR confirming AR mRNA levels in LNCaP and 22Rv1 cells with AR knockdown. **(f)** qRT-PCR analysis of relative *LTF* mRNA levels in 22Rv1 cells with AR knockdown. **(g)** qRT-PCR analysis of relative *LTF* and LTFe mRNA levels in prostate cancer cells treated with 100 nM DHT. **(h)** Enhanced interaction frequency between the enhancer and promoter of *LTF* in 22Rv1 cells treated with ENZ, determined by 3C assays. **(i-k)** Analysis of cell death rates (i), intracellular iron levels (j), and MDA levels (k) in 22Rv1 cells following treatment with DMSO, ENZ, or ENZ + sh*LTF*. **(l)** Viability of 22Rv1 cells pre-treated with 10 µM ENZ for 72 hours, then induced with varying concentrations of RSL3 for 24 hours. **(m)** Cell viability in 22Rv1 cell treated with RSL3 for 24 hour and enzalutamide for 72 hours at the indicated concentrations.

Data are presented as mean ± SD, n = 3 biologically independent replicates in (d), (e), (f), (g), (h), (i) or n = 5 biologically independent replicates in (j), (l), (m). Statistical analysis is performed using one-way ANOVA in (h), two-sided t test in (c), (d), (e), (f), (g), (i), (j).


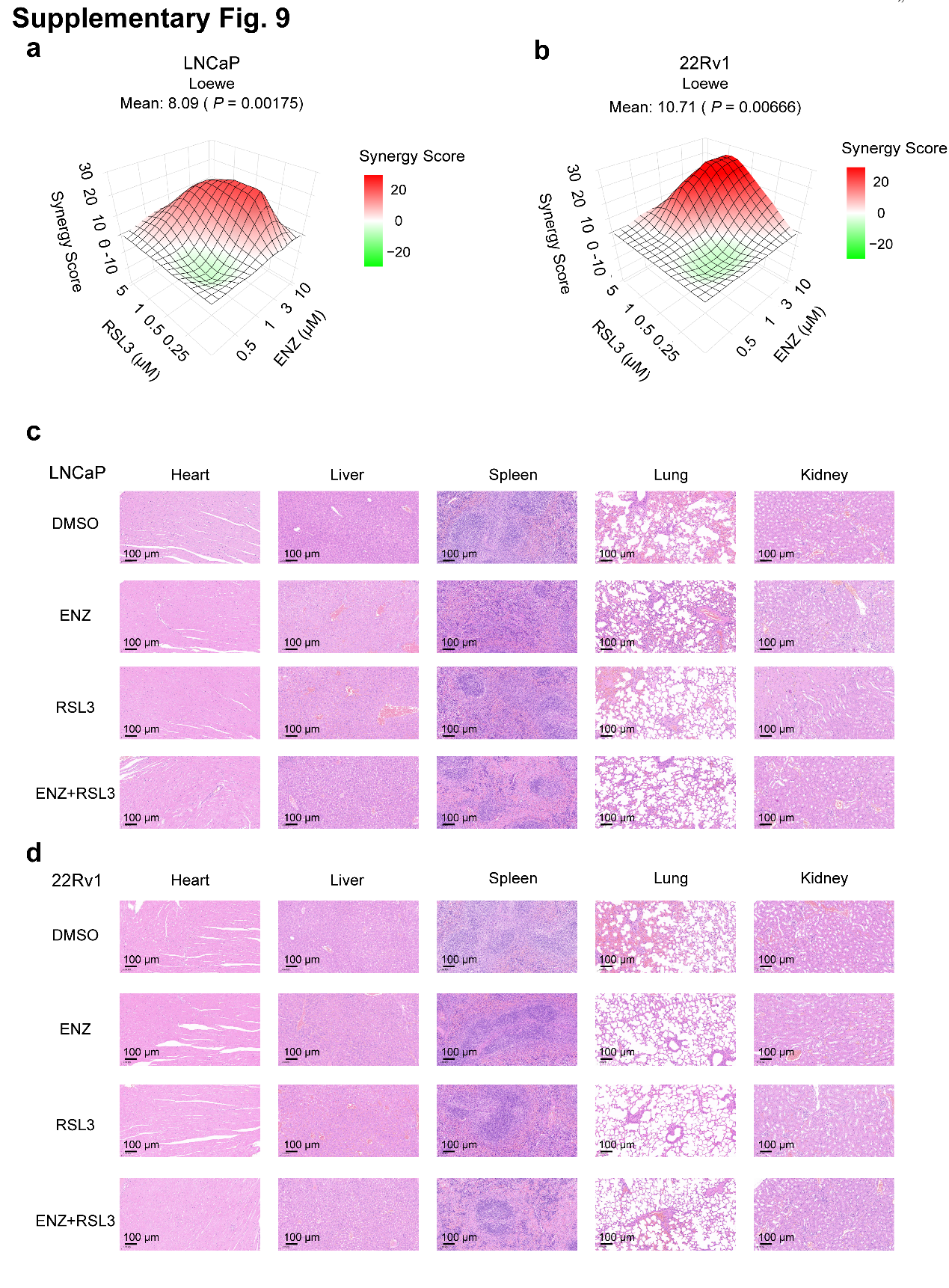


**Supplementary Fig. 9. Combining RSL3 and enzalutamide is an effective and safe treatment.**

**(a, b)** Loewe synergy score plots for LNCaP (a) and 22Rv1 (b) cells following the indicated combination treatments. Cells were pretreated with the specified concentration of RSL3 for 24 hours, followed by a 48-hour treatment with the indicated concentration of enzalutamide (ENZ). Synergy scores and plots were generated using SynergyFinder 3.0. **(c, d)** H&E staining of organs from nude mice in each treatment group. Scale bar = 100 μm.


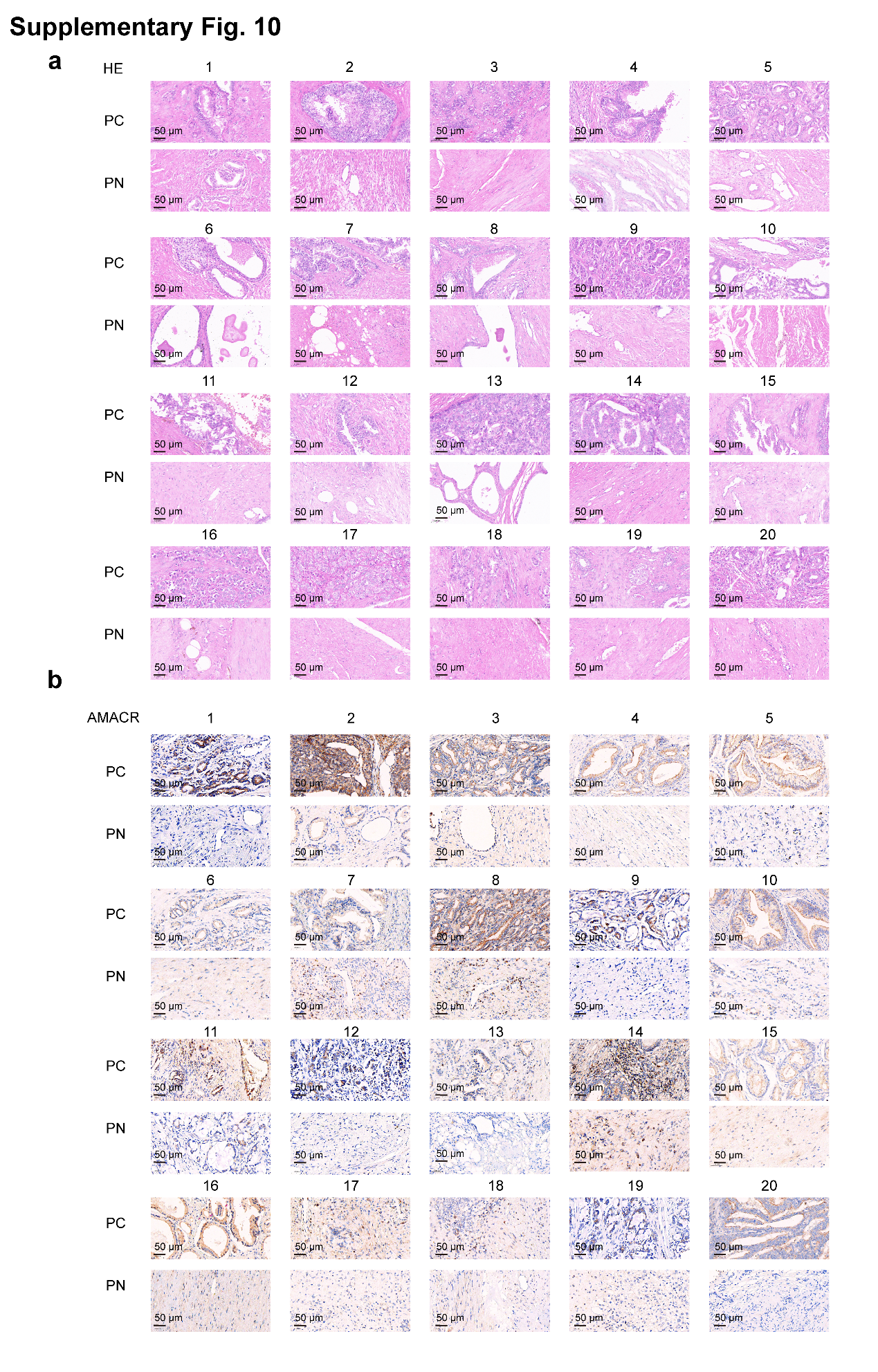


**Supplementary Fig. 10. Human samples used for ATAC-Seq and RNA-Seq****(a)** Hematoxylin and eosin (HE) staining of 20 pairs of prostate cancer and adjacent normal tissues. **(b)** AMACR immunohistochemistry of prostate cancer and adjacent normal tissues.

**Supplementary Table 1. The secondary structure of LTFe.**

| **The secondary structure of LTFe.** |
| --- |
| .......(((((((((((...(((((.((((((((((((....(((.(((((....(((((.....(((((.....((((((...........))))))....))))))))))(((((.......((((((...))))))........((((((.((.((....)).)).))).))).(((.((((((.((.((((((((.((.(((..(((((((..........))))))).))).)).)))))))(((((((((((((((((((((....(((((((((........((((((....((((((..(((.(((((((((((((.(.((((((((.....((.((((((((((((((......)))))).....((((((((((...(((.(((((((.....((((..((.(.(((((((..((((((((.(.....(((((((((.(((((((.........)))))))))).....((((......)))))))))).((((...)))).).)))))))).....((((.(.(((.....))).).)))).))))))).).))..))))....)))).)))..(((((((.((((...)))))))))))........)))...)))))))))))).)))).)).))....))))))))..).))..))))))((((......))))........................((((((((((.((.........)).))))...)))))).....))))))))..))))))..)))))).........))))))....(((((.....))))).(((((((((..........)))))))))..)))..))))))))(((((((((((..((((((..((((..(((((..(((((((...(((((((((....(((((.((((.....)))).)))))........((((((((((((........(((((..((((((((((.(((.(((((....)))...)).)))..)).)))).))))..))))).........)))).........))))))))..........))))..))))).)))))))..)).........)))...))))...))))...))..))))))))))).........)))))))))))))).)).))))))))))))))(((((..(((((((((..((.(((((..((.....))......))))).))..)))..))))))..)))))..((((((..((((((((((....)))).((.((((...)))))).((((((.((((((((((((((((((.((..(((...))).(((((((((((((....(((...(((((.((((((((((((........))))))))))))...))))).)))...((((((..((((..(((.((((((((.((.((.......)).))))))..))))))))))).))))))((((((((......((((((((....))))))))......)))).).))))))))).)).)))))...(((((((((((....(((...(((.((((((((((.(((((((....(((((..((.....((((((((.(((((.(((.....)))..)).))).)))))))).)))))))....(((.(((((.((((((.(((((..(((.((((((((((((((.......)))))..)))))))))))).))))).)))))).(((((((((.(((((..(((((..(.(((....((((((.((...(((.....(((((((((..(....).)))))))))....)))...)).))))))((((((..((((.((..(((((((...(((..((((.((.((((.(((((((((........((((((((..((.(((((((.((((.....(.((.(((((.......))))))))))))..))))).))((((((((((.....((((((((...(((((((((...((((((((((((.....)))))))(((.((((((.(((((((........(((.(((..((.((((.(.(((((.....))))))))))(((((((..((((((..((.(((((((...(((((....((((....((((.((((....(((((...((((((((((((......(((((((.(.(......(((((((..((((.....))))....)))).))).....).).)))))))))))))....))))))....((((.......))))((((((((((..(((......))).....))))).)))))..(((((((...((((((((((((.............(((((.....))))).(((((((.((.......(((((((.(((((...(((((((((((((((((((...((((.(((............))).))))...))))).((((..(((......)))...))))(((((.(((..((..(((((...((((((......((((((............................))))))..)))))).((....))((((((.........)))))).((((((((....))))))))...)))))..))...))).)))))))).)))))))).)))..)))))))))))))).)))))))...((((.((((((......((.(((((((((((....)))...)))))))).))...)))))).).))).......)))))))))))).))).))))))))).......)))).))))....))))...)))))..))))))).)).))).)))..)))))))...))))).)))))))))).)))))).))).....))))).)))..))))))..))))))))))))))))))((((.........(((((((.((.(((..........))).)))))))))......))))..........(((((((((((..((((((((((((...((((((((.......((((.(((((.((.((((...((((((.((((((.(.(((......))).).))))..)).)))))))))).)).........(((........)))((((((........))))))))))))))).......))))))))...)))))))))).........((((....(((((..(((((((((...(....)...))))).)))).)))))..))))......((((((((((((((...((.(((..((((.......)))).))))).))))))...)))))))).....(((...((....))...))).))..)))))))).)))))))))))))...))))))))))))).)).).))))))))))))))).))))))))))..(((........))).))).)..)))))..)))))....))))))))).........((((((.........((((((..((((((..((((....))))...))))))))))))......))))))..........))))).)))(((...(((((((((((.....((........)).......)))))))).)))...)))))))))).)))))))))).))).)))(((((((.((((((((.......((((((((((((.(((((((((((((.....))))))))))).)))))).......(((((((((((((((((((.((((...(((((......(.........)......))))).....)))))))))))))))))))))))(((((.((.(((..((((((((.(((..(((((((.......))))..)))..)))))))))))(((.((((((((.(((...)))))))).)))))).....))))).)))))((((.(((....))))))).............)))))))).....)))))).)).)))))))..(((((........(((((((((.(......).)))))))))......)))))...(((((..((.(((.(((((((.((((..((((((...........))))))))))...((((.......)))).......(((((((.....)))))))............(((((((((((.(((..(.((((....).))))..))).))))).))))))))))))).))))).))))))))).....)))))))......(((((.......(((((((((..........................)))))))))((((.((....))..))))((((((((..((((.((((((((((((((((.........))))(((((.(((((((.....))).)))).))))).(((((..(((((..((((((((((((((.........((((.....)))).........))))).))))))))).(((((((((..(((((.........)))))...........((((...))))..........................))))))))).(((((((.((((((((((((((.........)).)))))))..))))))))...))))....((((.....))))((((((..(....)..))))))....((((((((......))))))))(((((...................))))).(((((.(((....((((((((....))))))))))).)))))..))))).)))))....)))))).............))))))))))..))).))))).......)))))))))))))))))))))...(((((.(.((((((.((((((((((((....))))))))....((((((...(((((.....(((.(((......))).)))......)))))))))))......(((((.....)))))..(((...((....(((((....)))))))....))))))).))))))))))))))))))))))........))))))..)))))).))))))))...))))))))))))((((((((.((................)).))))))))........((((((((......(((........)))((((((...((((.(((..(((((...)))))...))).)))).....))))))..(((((((.(((((((((..(((((..(((.....((((((...)))))).....)))..)))))..))))))))).))))))).(((((((((((((....))..))))))))))).....(((((((((((((((((((((...))))(((..((((.((..((((((((..(((.........)))...))))..))))....)).)))).)))((((..(.((((((((.((...(((..((((((((......(((....)))......)))))))).......))).....)).)))))))))..))))......(((((...)))))..((.(((((((....(((((.......)))))..(((((((..((((((.(((((.(((((((......))))))).((.((((...((((......))))....)))).)).....((((((((........((((......))))........)).))))))....((((((((..(((.......((((((.(...((((((.(....).))))))...)...)))))).....)))))))))))........))))).)))))).))).))))..(((((.....)))))......(((((.......)))))))))))).))...)))))))))))))))))))))))))..(((((.........)))))....)))))....)))))))..((((...((((....)))).....)))).)))). |

**Supplementary Table 2. Human samples used for ATAC-seq and RNA-seq**

| **ID#** | **Age (years)** | **PSA (ng/ml)** | **TNM stage** | **Gleason score** |
| --- | --- | --- | --- | --- |
| 380 | 64 | 18.47 | T2N0M0 | 4+4=8 |
| 385 | 76 | 28.24 | T3aN0M0 | 4+5=9 |
| 386 | 68 | 27.45 | T3aN0M0 | 4+3=7 |
| 546 | 62 | 14.90 | T3aN0M0 | 4+4=8 |
| 763 | 65 | 24.76 | T3bN0M0 | 3+4=7 |
| 866 | 74 | 12.95 | T3aN0M0 | 4+3=7 |
| 876 | 75 | 17.67 | T3bN0M0 | 3+4=7 |
| 895 | 67 | 44.41 | T4N0M1 | 4+5=9 |
| 915 | 62 | 78.25 | T3aN0M0 | 3+4=7 |
| 1056 | 73 | 54.89 | T4N0M0 | 3+4=7 |
| 1462 | 63 | 12.66 | T2aN0M0 | 3+4=7 |
| 1464 | 66 | 14.43 | T2aN0M0 | 3+4=7 |
| 1522 | 65 | 3.25 | T2aN0M0 | 3+4=7 |
| 1537 | 68 | 56.08 | T2bN0M0 | 4+3=7 |
| 1549 | 64 | 21.34 | T3bN0M0 | 4+5=9 |
| 1568 | 70 | 4.65 | T2bN1M0 | 3+4=7 |
| 1590 | 63 | 0.05 | T1N0M0 | 3+3=6 |
| 1603 | 77 | 37.87 | T3bN1M0 | 4+4=8 |
| 1612 | 76 | 148.26 | T4N1M0 | 4+3=7 |
| 1619 | 71 | 13.74 | T3bN0M0 | 4+3=7 |

**Supplementary Table 3. Characteristics of patients in Tongji PCa cohort**

| Sample lD | Age (year) | Gleason | T | N |
| --- | --- | --- | --- | --- |
| 1 | 59 | 6 | T2c | N0 |
| 2 | 74 | 8 | T3a | N0 |
| 3 | 57 | 6 | T2a | NX |
| 4 | 52 | 7 | T3a | N0 |
| 5 | 58 | 7 | T4 | N1 |
| 6 | 62 | 7 | T3b | NX |
| 7 | 67 | 8 | T3b | N0 |
| 8 | 68 | 7 | T2 | N1 |
| 9 | 67 | 9 | T4 | N1 |
| 10 | 69 | 7 | T2c | NX |
| 11 | 55 | 7 | T2c | N0 |
| 12 | 74 | 9 | T3b | N0 |
| 13 | 70 | 7 | T2 | N0 |
| 14 | 62 | 7 | T2b | N1 |
| 15 | 72 | 7 | T2a | N0 |
| 16 | 62 | 7 | T3b | NX |
| 17 | 55 | 7 | T2c | N0 |
| 18 | 62 | 7 | T2b | N0 |
| 19 | 51 | 6 | T2a | N0 |
| 20 | 59 | 8 | T2 | N0 |
| 21 | 56 | 7 | T3a | NX |
| 22 | 65 | 8 | T4 | NX |
| 23 | 67 | 8 | T3a | N1 |
| 24 | 70 | 9 | T2c | N0 |
| 25 | 66 | 8 | T3b | NX |
| 26 | 52 | 7 | T2a | NX |
| 27 | 64 | 7 | T2 | N0 |
| 28 | 71 | 7 | T2a | NX |
| 29 | 68 | 7 | T2c | NX |
| 30 | 65 | 9 | T3b | N1 |
| 31 | 73 | 7 | T2a | NX |
| 32 | 59 | 6 | T2a | N0 |
| 33 | 67 | 6 | T2a | NX |
| 34 | 71 | 7 | T4 | N1 |
| 35 | 61 | 7 | T4 | N0 |
| 36 | 73 | 7 | T2b | N0 |
| 37 | 57 | 6 | T3a | N0 |
| 38 | 60 | 7 | T3a | N0 |
| 39 | 68 | 7 | T4 | N1 |
| 40 | 57 | 9 | T4 | N0 |
| 41 | 73 | 7 | T4 | N0 |
| 42 | 59 | 8 | T4 | N0 |
| 43 | 78 | 7 | T2c | N0 |
| 44 | 75 | 9 | T2a | NX |
| 45 | 54 | 7 | T4 | N1 |
| 46 | 62 | 8 | T4 | N1 |
| 47 | 62 | 6 | T2b | NX |
| 48 | 51 | 8 | T3b | N1 |
| 49 | 67 | 9 | T4 | N1 |
| 50 | 70 | 7 | T2b | N0 |
| 51 | 76 | 6 | T2a | NX |
| 52 | 63 | 9 | T2b | N0 |
| 53 | 81 | 9 | T4 | N1 |
| 54 | 56 | 7 | T3a | NX |
| 55 | 59 | 7 | T2 | N0 |
| 56 | 66 | 8 | T3b | NX |
| 57 | 64 | 8 | T4 | N1 |
| 58 | 70 | 8 | T2a | NX |
| 59 | 65 | 8 | T3b | N1 |
| 60 | 72 | 8 | T4 | N1 |
| 61 | 68 | 7 | T3b | NX |
| 62 | 67 | 9 | T4 | NX |
| 63 | 71 | 6 | T2a | N0 |
| 64 | 74 | 8 | T3b | Nx |
| 65 | 72 | 9 | T3a | N1 |
| 66 | 62 | 7 | T2a | NX |
| 67 | 59 | 7 | T2b | N0 |
| 68 | 75 | 8 | T2c | N0 |
| 69 | 66 | 7 | T3b | N0 |
| 70 | 76 | 8 | T3b | N0 |
| 71 | 67 | 9 | T4 | N1 |
| 72 | 74 | 9 | T3b | NX |
| 73 | 67 | 9 | T3b | N0 |
| 74 | 64 | 9 | T4 | N1 |
| 75 | 67 | 8 | T3b | N0 |
| 76 | 66 | 6 | T2a | N0 |
| 77 | 66 | 7 | T2 | N0 |
| 78 | 58 | 7 | T3b | N1 |
| 79 | 56 | 9 | T4 | N1 |
| 80 | 63 | 7 | T2a | N0 |
| 81 | 67 | 6 | T3a | NX |
| 82 | 77 | 8 | T3b | Nx |
| 83 | 57 | 7 | T2a | NX |
| 84 | 77 | 9 | T3a | N0 |
| 85 | 62 | 9 | T3a | NX |
| 86 | 73 | 8 | T4 | NX |
| 87 | 59 | 7 | T3a | N0 |
| 88 | 60 | 8 | T4 | N1 |
| 89 | 70 | 7 | T2b | N0 |
| 90 | 60 | 6 | T2 | N0 |
| 91 | 60 | 7 | T3b | NX |
| 92 | 53 | 7 | T2a | NX |
| 93 | 65 | 7 | T3a | NX |
| 94 | 75 | 9 | T4 | NX |
| 95 | 77 | 8 | T4 | N0 |
| 96 | 79 | 8 | T2a | NX |

**Supplementary Table 4. The quality control profile of our ATAC-seq data.**

| **Sample** | **Raw Reads** | **Raw Bases(G)** | **Raw Q20(%)** | **Raw Q30(%)** | **Raw GC(%)** | **Clean Reads** | **Clean Bases(G)** | **Clean Q20(%)** | **Clean Q30(%)** | **Clean GC(%)** | **Effective Rate(%)** |
| --- | --- | --- | --- | --- | --- | --- | --- | --- | --- | --- | --- |
| PC1 | 1.05E+08 | 15.75 | 91.03 | 83.41 | 52.60 | 1.04E+08 | 8.47 | 97.31 | 92.17 | 44.89 | 98.96 |
| PC2 | 1.04E+08 | 15.62 | 91.59 | 84.28 | 52.68 | 1.03E+08 | 8.75 | 97.44 | 92.46 | 45.49 | 99.02 |
| PC3 | 1.61E+08 | 24.14 | 94.83 | 89.82 | 49.14 | 1.59E+08 | 17.09 | 97.68 | 93.48 | 45.20 | 98.59 |
| PC4 | 1.03E+08 | 15.51 | 92.01 | 84.47 | 49.95 | 1.03E+08 | 9.50 | 97.41 | 92.02 | 42.71 | 99.25 |
| PC5 | 1.05E+08 | 15.68 | 91.47 | 83.72 | 51.93 | 1.04E+08 | 8.93 | 97.23 | 91.81 | 44.13 | 99.06 |
| PC6 | 1.06E+08 | 15.97 | 96.19 | 91.09 | 49.88 | 1.05E+08 | 9.37 | 98.00 | 94.03 | 44.16 | 99.56 |
| PC7 | 1.03E+08 | 15.62 | 95.89 | 90.66 | 50.00 | 1.03E+08 | 10.30 | 97.89 | 93.86 | 45.95 | 99.41 |
| PC8 | 1.07E+08 | 16.09 | 95.57 | 89.99 | 51.71 | 1.06E+08 | 8.88 | 97.91 | 93.83 | 45.55 | 99.49 |
| PC9 | 1.06E+08 | 16.00 | 95.48 | 90.01 | 53.40 | 1.05E+08 | 7.77 | 98.17 | 94.47 | 45.50 | 99.44 |
| PC10 | 1.07E+08 | 16.08 | 93.65 | 88.07 | 51.99 | 1.06E+08 | 8.06 | 98.16 | 94.49 | 44.75 | 99.54 |
| PN1 | 1.04E+08 | 15.65 | 92.30 | 84.92 | 50.04 | 1.03E+08 | 9.46 | 97.34 | 92.05 | 43.98 | 99.01 |
| PN2 | 1.06E+08 | 15.87 | 90.37 | 82.41 | 54.96 | 1.04E+08 | 7.29 | 97.39 | 92.53 | 47.34 | 98.75 |
| PN3 | 1.06E+08 | 15.86 | 90.79 | 83.19 | 53.90 | 1.05E+08 | 7.32 | 97.55 | 92.89 | 44.78 | 98.98 |
| PN4 | 1.04E+08 | 15.67 | 91.85 | 84.26 | 50.89 | 1.04E+08 | 8.92 | 97.50 | 92.29 | 43.39 | 99.29 |
| PN5 | 1.05E+08 | 15.74 | 90.52 | 82.95 | 52.08 | 1.04E+08 | 8.63 | 97.36 | 92.32 | 46.30 | 98.94 |
| PN6 | 1.06E+08 | 15.99 | 95.76 | 90.42 | 52.39 | 1.06E+08 | 7.48 | 98.27 | 94.67 | 44.61 | 99.63 |
| PN7 | 1.04E+08 | 15.68 | 95.50 | 89.97 | 50.28 | 1.03E+08 | 9.04 | 98.07 | 94.18 | 44.32 | 99.56 |
| PN8 | 1.16E+08 | 17.48 | 95.20 | 89.35 | 53.36 | 1.15E+08 | 8.11 | 98.17 | 94.39 | 45.81 | 99.61 |
| PN9 | 1.06E+08 | 15.95 | 95.53 | 90.03 | 53.64 | 1.05E+08 | 7.89 | 98.18 | 94.52 | 47.44 | 99.43 |
| PN10 | 1.06E+08 | 16.01 | 93.04 | 87.21 | 55.18 | 1.05E+08 | 6.98 | 98.26 | 94.79 | 48.64 | 99.27 |
| PC11 | 1.19E+08 | 17.79 | 95.32 | 89.50 | 46.38 | 1.18E+08 | 12.84 | 97.26 | 92.30 | 42.10 | 99.20 |
| PC12 | 1.17E+08 | 17.53 | 94.85 | 89.00 | 49.31 | 1.16E+08 | 11.84 | 97.35 | 92.49 | 45.38 | 99.22 |
| PC13 | 1.18E+08 | 17.66 | 95.41 | 89.43 | 47.79 | 1.16E+08 | 13.50 | 96.84 | 91.43 | 45.13 | 98.90 |
| PC14 | 1.06E+08 | 15.86 | 94.69 | 88.99 | 48.24 | 1.05E+08 | 11.66 | 97.08 | 91.94 | 44.44 | 99.04 |
| PC15 | 1.01E+08 | 15.18 | 94.66 | 88.79 | 48.97 | 1.01E+08 | 8.71 | 97.88 | 93.65 | 42.17 | 99.49 |
| PC16 | 1.72E+08 | 25.78 | 95.12 | 89.12 | 48.78 | 1.71E+08 | 17.23 | 97.32 | 92.40 | 44.48 | 99.32 |
| PC17 | 1.18E+08 | 17.69 | 94.47 | 88.66 | 49.12 | 1.17E+08 | 11.58 | 97.57 | 92.94 | 44.63 | 99.41 |
| PC18 | 1.46E+08 | 21.83 | 95.42 | 89.72 | 49.04 | 1.45E+08 | 14.41 | 97.51 | 92.85 | 44.71 | 99.34 |
| PC19 | 98458634 | 14.77 | 95.35 | 89.24 | 47.37 | 97658848 | 10.40 | 96.97 | 91.60 | 43.09 | 99.19 |
| PC20 | 1.3E+08 | 19.53 | 95.53 | 89.71 | 47.97 | 1.29E+08 | 13.21 | 97.42 | 92.63 | 44.04 | 99.31 |
| PN11 | 1.26E+08 | 18.93 | 94.55 | 88.72 | 47.95 | 1.25E+08 | 12.76 | 97.45 | 92.67 | 43.20 | 99.37 |
| PN12 | 1.32E+08 | 19.87 | 95.57 | 89.82 | 48.27 | 1.32E+08 | 13.34 | 97.44 | 92.67 | 44.22 | 99.30 |
| PN13 | 1.26E+08 | 18.96 | 93.96 | 88.31 | 50.81 | 1.26E+08 | 12.03 | 97.62 | 93.13 | 46.00 | 99.31 |
| PN14 | 1.41E+08 | 21.18 | 95.24 | 89.64 | 49.72 | 1.4E+08 | 13.56 | 97.61 | 93.15 | 45.11 | 99.25 |
| PN15 | 1.07E+08 | 16.07 | 95.33 | 89.44 | 46.77 | 1.07E+08 | 10.36 | 97.52 | 92.78 | 40.73 | 99.50 |
| PN16 | 1.03E+08 | 15.48 | 93.81 | 87.98 | 49.66 | 1.02E+08 | 10.21 | 97.30 | 92.43 | 44.85 | 99.17 |
| PN17 | 1.12E+08 | 16.81 | 95.36 | 89.77 | 49.63 | 1.11E+08 | 11.12 | 97.55 | 92.98 | 45.61 | 99.27 |
| PN18 | 1.16E+08 | 17.34 | 94.82 | 88.66 | 48.48 | 1.15E+08 | 11.36 | 97.25 | 92.18 | 43.44 | 99.39 |
| PN19 | 1.24E+08 | 18.53 | 92.93 | 87.23 | 51.26 | 1.23E+08 | 11.31 | 97.51 | 92.83 | 44.94 | 99.34 |
| PN20 | 1.13E+08 | 16.90 | 94.43 | 88.71 | 48.13 | 1.12E+08 | 11.57 | 97.48 | 92.73 | 43.58 | 99.34 |

**Supplementary Table 5. Probes or primers sequence used in the study**

| **ASOs** | | |
| --- | --- | --- |
| LTFe | ASO-1 | GAACTTTCAATTCTGTCTGC |
|  | ASO-2 | ACGTGAGCTGTGAGGAAACT |

| **RT-qPCR (5’-3’)** | | |
| --- | --- | --- |
| GAPDH | Forward | CTGGGCTACACTGAGCACC |
|  | Reverse | AAGTGGTCGTTGAGGGCAATG |
| U1 | Forward | ACTTACCTGGCAGGGGAGATACC |
|  | Reverse | CCACTACCACAAATTATGCAGTCG |
| LTFe | Forward | GAGAGACTCCCCCATCCAGT |
|  | Reverse | GGGAGCCAAAGAGCTGGAAT |
| LTF | Forward | CCCAGGAACCGTACTTCAGC |
|  | Reverse | GTGCCACAACGGCATGAGA |
| HNRNPF | Forward | ACTGCCAGGAGGTACATTGG |
|  | Reverse | CTGAGGTCTCTCCCGAACAG |
| AR | Forward | GTGGAAGCTGCAAGGTCTTC |
|  | Reverse | TTCAGATTACCAAGTTTCTTCAGC |

| **shRNAs** | | |
| --- | --- | --- |
| shLTF | shRNA-1 | CCTGATCCTAACTGTGTGGAT |
|  | shRNA-2 | CCCTACAAACTGCGACCTGTA |
| shHNRNPF | shRNA-1 | CGAGAACGACATTTACAACTT |
|  | shRNA-2 | GAAGGCTCTAGGGAAACACAA |
| shAR | shRNA-1 | GGACACTTGAACTGCCGTCTA |
|  | shRNA-2 | CCTGCTAATCAAGTCACACAT |

| **ChIP-qPCR (5’-3’)** | | | |
| --- | --- | --- | --- |
| LTF-promoter | 1 | Forward | GGTCCTCAATTCCACCAGCA |
|  |  | Reverse | TCGGCTGAGGGGAAGTTTTC |
|  | 2 | Forward | TCCTGGTTAGCTTAGGGGCT |
|  |  | Reverse | ATAGCCAAGGCCTGCTTGTT |
|  | 3 | Forward | GCCAAACTTGCGCCTTTACA |
|  |  | Reverse | TCGGGGTCTCTGAGTCCATT |
|  | 4 | Forward | GGAGGCTGGGACAAATGACA |
|  |  | Reverse | TCCCATCCACCTCTCATCCA |
| LTF-enhancer | 1 | Forward | CTCTACCACATGCAGCCACA |
|  |  | Reverse | CACTAGTCCTGGGTGCAGTG |
|  | 2 | Forward | CTCTACCACATGCAGCCACA |
|  |  | Reverse | CTAGTCCTGGGTGCAGTGTG |
|  | 3 | Forward | AGGTAAGATGGCTGGGGGAT |
|  |  | Reverse | TAGGGTAACGGATCCTGCCA |
|  | 4 | Forward | TGCTGGGTTTGGGTGAGTTT |
|  |  | Reverse | AACTCACGTCTTTCGGTCCC |
|  | 5 | Forward | AGAATAACTCAAGGCCGGGC |
|  |  | Reverse | CCTCCTGAGTAGCTGGGACT |

| **RIP-qPCR (5’-3’)** | | | | | |
| --- | --- | --- | --- | --- | --- |
| LTFe | 1 | | Forward | | CACTGCACCCAGGACTAGTG |
|  |  | | Reverse | | TGGTGGCCTCTGATGAATGC |
|  | 2 | | Forward | | CAGACCCCGCATTCATCAGA |
|  |  | | Reverse | | ATTCCAGACAGCAGCTTGGT |
|  | 3 | | Forward | | CCTGTGCTTACCCCTGATGG |
|  |  | | Reverse | | CGGCTCATTACCCTGCTCTT |
|  | 4 | | Forward | | GAATGATAGCCTGCCCTGCT |
|  |  | | Reverse | | CCCAGATGAGGAGCACGTTT |
| **Probes for ChIRP** | | | | | |
| Odd | | set1 | | gtcgtttgttctagatactt | |
|  |  | set2 | | aggacctcaaaaagtctcca | |
|  |  | set3 | | cattccctacatgtgtgatg | |
|  |  | set4 | | gcagttcgttcagctgaaag | |
| Even | | set1 | | tgttttgggacagtgagcac | |
|  |  | set2 | | atgaaaccaccatcaagggt | |
|  |  | set3 | | aacccatcctgaaaagtctc | |
|  |  | set4 | | cctgtgcttacaactggaat | |
| LacZ | | set1 | | TCACGACGTTGTAAAACGAC | |
|  |  | set2 | | GGTTACGTTGGTGTAGATG | |
|  |  | set3 | | GTAGCCAGCTTTCATCAACA | |
|  |  | set4 | | AGATGAAACGCCGAGTTAAC | |

| **Chromosome conformation capture assay (5'-3')** | | |
| --- | --- | --- |
| GAPDH | Forward | ACAGTCCATGCCATCACTGCC |
|  | Reverse | GCCTGCTTCACCACCTTCTTG |
| Cut | Reverse | TGGCAGGATCCGTTACCCTA |
| Cut site1 | Forward | ATTTAGGCGCCTTCAGGACC |
| Cut site2 | Forward | ATTCTGCCAGGAACTTGGGG |
| Cut site3 | Forward | AGAACTCGACAGTGAAACCA |
| Cut site4 | Forward | GGCTTCCTCTTTCCCATGCA |
| Cut site5 | Forward | TCACTGGAAATGGCAGGAGC |
| Cut site6 | Forward | GATATCCACGTGTGCTCCCA |
| Cut site7 | Forward | TCATCCATGCTGAGCTGCAA |
| Cut site8 | Forward | GGCTTCACCTCCCGCATTAA |
| Cut site9 | Forward | TGGTGCAATGCTCCCTATGG |
| Cut site10 | Forward | TCTGAATACTGCACTGGGCC |
| **Sequence used for LTFe overexpression (5'-3')** | | |
| CAGTAAGAGGGAATTCATAATTAATAAAAAATACTTGTGAGATGTTCCCCTTACTAATCAGACCTGAGCGATTTGTTAGTACTCACATTTTCTATAGTAATAACAGCTAATAGAATGATAGCCTGCCCTGCTGGTGATTCAAGAGTGAAGCTGGTCCTCACGGTCAGCGCCATTCTGAATTGGTATTATAAGTCTTCTGGAAGCAGAATGGCAACGGCAGCCCGGGTCACAGGGGGAAACGTGCTCCTCATCTGGGGGATTCCACCCTCAGCTGCCTACCCAAGGACACAAGTGGTCACAAGGAAAAACACTGAGGACAAAGATATTCATGAAAATACAGTCTTCACAGGAAACAGTTAGAAATAAACTTAAATGCTAAACATTGGGGGAAATTATTCAATACACGATAGAACACCCACATTATGAGAAAACATTATGCTGTCTACAAAATCAGTAAAGGCTCTGCAGAAGTTAGAAAATGTATCCAGTTTTAGAGGAATAAGCAGTCCACAAAATATGATGTATCCCAGGGGTCCCCACCACCTGGCCACGGACTGGTACCGGTTGATGGCCTGTTAGGAACTAGACCTCAGACCAGGAGGTGAGCAGAGGGCCAGTGAGCATGACTGCCTGAGCTCCACCTCCTGTCAGATCAGCGGCGGCATTAGATTCTCATAGGAGTGTGAACCCTAGTGTGAACTGTGCATGCGAGGGATCTAGGTTGCACTCCCTATGAGAATCCAATGCCTGATGATCAGAGGTGGAACGGTTTCATCCCAAAACCATTCCCCATCTGTCTGTGGAAAAATTACATTCCCTGAAACTGGTCCTTGGTGGCAAAAAAGTTGGGGACCACTAAACTATCCCACATTGCAAACCAAAGAAATATGTAATCATGTGGGAAGCAATGTGCTGAAGCGGAGGAACATGGTTTCTTATGAAAATTTTAGGATAGCTTAATAACTATATTTTCTATCCAATAAAACAGAACTTAAAAGAAATGAATATAGAGCAAAAATAGAAAGTATCTAGAACAAACGACTTCAAATATAGATGACTGTGAGGGGTGTGATCGGGAGAGTGACACAGGCAGGCAGAGGAGAAAGAGGGCAGGCGCCAGTCTCAGGACTTAGAGGGCTGGTGCTCACTGTCCCAAAACAGGGGTCCTTGGCTCTCACCATCTCTCATAGGAAGTGAGGGGACAGGATGGAAAGCGGACCCCTTTGACGACCCTTCCAGCCATGGAGACTTTTTGAGGTCCTGAAGTCCCACTTGCTGGGTTTGGGTGAGTTTTCTGCTGAAGCCAGTCTGGCCTCTTTACTTTCAGGAAAACAGGGCCGATGCTGTGACCCTTGATGGTGGTTTCATATACGAGGCAGGCCTGGCCCCCTACAAACTGCGACCTGTAGCGGCGGAAGTCTACGGGACCGAAAGACGTGAGTTCTGCCTGGGGACCCAGAGGCCACGGTGGCCTCAGCCTGTGCCCTGAGCTGTGTGGATTAAGACTGGGGGAACATGTGGAGGTGGAGTCTGGGTCACATCACACATGTAGGGAATGGAGTCGCTGGGCTCTGGGCCAGATGAAGGCCGTTCCTCCTGACGCTGACCCACGAGAGGAGGACACACGTGAGCTGTGAGGAAACTGCAGCACAGCATTCCCCCTTCCCACCGGAGACTTTTCAGGATGGGTTGTTTTTGTCCCTCTTGTCCAGGCTAAGAACTTTCAATTCTGTCTGCCCCTTTGCAGAGCCACGAACTCACTATTATGCCGTGGCTGTGGTGAAGAAGGGCGGCAGCTTTCAGCTGAACGAACTGCAAGGTCTGAAGTCCTGCCACACAGGCCTTCGCAGGACCGCTGGATGGAATGTCCCTATAGGGACACTTCGTCCATTCTTGAATTGGACGGGTCCACCTGAGCCCATTGAGGCAGGTAAGATGGCTGGGGGATAGTGAGTGGCCTCAGGCAGGGGGCTCTATTCCAGTTGTAAGCACAGGCCACACAGATCATGCAGGTGAAAGTGTGGGATGAATCAAGGTGGGGGTGAGGCTGGCCAGCTTGTAACATCCTGCTGGCAGGATCCGTTACCCTAGCAGCCCTTGGGAGGCACAGCTGAGTCTGCTCTCGGCAGAGGTGCATGTCTCGAGCTCCCAGCCCCATGACAGAGTCTCTCCTGCAGGGGTGGAGGAAGGGGCCTTGCCCACGGAGACCTCAGGATGGGAGGTGTAACCTGCTGTGACCAGGGCTGGCTCACACTCTGTGGTCCACTTCTCTGTGTTTAACAGCTGTGGCCAGGTTCTTCTCAGCCAGCTGTGTTCCCGGTGCAGATAAAGGACAGTTCCCCAACCTGTGTCGCCTGTGTGCGGGGACAGGGGAAAACAAATGTGCCTTCTCCTCCCAGGAACCGTACTTCAGCTACTCTGGTGCCTTCAAGTGAGTGACCCTGTCCCCTTCTCGTCAGTGGCCAAGTGTCCCTTGGCCTCAGGCCGGGAGGCCTTTTCTCTGGCCCCACATAGAGCCCAGCCTGCTCTTGGGGACGAGAGGAGGTCTGTTCTCCTACTGCTGTGTGTCCAAAGAGAGTGCAGGCCTGCCCAGTGTGTGTTCCCCTCCAGCCTTCCGGGCCCAACTGTGCCCCCAACTTCTCACCAGCCCCACGGGCAGTCACTGTGGCTGTGGGCCCTCCTGTTACCTCAGAGAGCCCTGAATCCAACTTGGCTGCCCTGTTGTCCTGGGTTCCTCATACTAATTTCTGCCGCCTGCCTAACCACAGCAGGACTGAAGGCATCTTGTCATGAGATGTCCAGTCCTGGCTCCTGGTTGGGCAGGACCGCTGCAGTGTCCAGGCTGATGTCCTTCTGCCCACCTGGGCCTCTCCCCGTGGCTGAAGGACAGTGAGCAATGCCTGATTCGCCCCCATCCTCTCTGCCCCCACGCGGAGGCTGAGCTCCCCCTTTTCCCATTCTGTCTGCTGGCACTAGCGTATTTTTGCAGAGGGAGGCCTCC | | |

**Supplementary Table 6 Antibodies used in the research**

| Antibodies | Source | Identifier |
| --- | --- | --- |
| H3K27ac | Cell Signaling Technology | #8173 |
| H3K4Me1 | Cell Signaling Technology | #5326 |
| H3K4Me3 | Cell Signaling Technology | #9751 |
| LTF | Abcam | ab109216 |
| HNRNPF | Proteintech | 67701-1-Ig |
| β-actin | ABclonal | AC038 |
| MDA | Abcam | ab27642 |

Data S1. (separate file)

Data S1: The enhancer transcription profile
